# Molecular Characterization and Expression Profiles of Polygalacturonase Genes in *Apolygus lucorum* (Hemiptera: Miridae)

**DOI:** 10.1371/journal.pone.0126391

**Published:** 2015-05-08

**Authors:** Lili Zhang, Pengjun Xu, Haijun Xiao, Yanhui Lu, Gemei Liang, Yongjun Zhang, Kongming Wu

**Affiliations:** State Key Laboratory for Biology of Plant Diseases and Insect Pests, Institute of Plant Protection, Chinese Academy of Agricultural Sciences, Beijing, China; USDA-ARS, UNITED STATES

## Abstract

Polygalacturonase (PG) is an enzyme in the salivary glands of piercing-sucking mirid bugs (Hemiptera: Miridae) that plays a key role in plant feeding and injury. By constructing a full-length cDNA library, we cloned and characterized 14 PG genes from the salivary glands of *Apolygus lucorum*, a pestiferous mirid bug in cotton, fruit trees and other crops in China. BLAST search analysis showed that the amino acid sequences deduced from transcripts of the PG genes were closely related to PGs from other mirid bugs. Phylogenetic analysis showed that the PGs of mirid bugs had six main branches, PG1-PG6 (Genbank accession numbers: KF881899~KF881912). We investigated the mRNA expression patterns of the *A*. *lucorum* PG genes using real-time PCR. All 14 PGs were expressed significantly higher in the salivary glands than in other tissues (head, thorax, abdomen, leg and wing). For eggs and nymphs, the expression levels of these PGs were much higher in the 5^th^ instar stage than in the egg, and 1^st^ and 3^rd^ instar stages. The PG expression levels in 1-day-old adults were very low, and increased in 5, 20 and 30-day-old adults. Additionally, PG expression levels were generally similar between males and females. The possible physiological functions of PGs in *A*. *lucorum* were discussed.

## Introduction

The mirid bug *Apolygus lucorum* (Meyer-Dür) (Hemiptera: Miridae) is a polyphagous insect pest with more than 150 described host plants [1,2]. In China, *A*. *lucorum* has been regarded as a secondary crop pest throughout the past century and has not usually required specific management in crop production [3]. Since the late 1990s, the wide-scale adoption of insect-resistant transgenic cotton in China has effectively suppressed the target pest, *Helicoverpa armigera* (Hübner), and greatly reduced the associated pesticide input for this crop. As a result, the population levels of *A*. *lucorum* and *Adelphocoris* spp. have drastically increased, resulting in serious yield losses in cotton and many other neighboring host crops (mainly Chinese dates, cherries, grapes, apples, pears, and tea) [3,4]. Similar to other species of mirid bugs (Hemiptera: Miridae), both *A*. *lucorum* nymphs and adults feed on plant tissues using piercing and sucking mouthparts. As its stylet penetrates (probes), saliva containing several digestive enzymes is introduced into the target tissues in a "lacerate and flush" action [2,5,6]. This type of feeding damages plants and is responsible for the stunting, abscission of squares and bolls (in cotton), and fruit malformation of plants [2,7]. Our previous study indicated that saliva, rather than the mechanical damage caused by stylet probing, was the key factor eliciting feeding-damage symptoms from *A*. *lucorum* [8]. However, the mechanisms of the formation of plant injury elicited by *A*. *lucorum* feeding, especially the role of its salivary enzymes in this physiological process, have not yet been determined.

Among the various digestive enzymes in the salivary glands of mirid bugs, polygalacturonase (PG) is one of the most important in the induction of visible plant injury. This enzyme catalyzes hydrolysis of the α-1,4-glycosidic linkages in polygalacturonic (pectic) acid in plant cell walls. PG characteristics and function have been extensively studied in *Lygus* spp., an important group of pest mirid bugs in cotton, strawberry, alfalfa, beans and other crops in northern America and Europe. In 1968, Strong and Kruitwagen [9] documented the presence of a potent PG in lygus salivary glands. Later, Strong [7], noting that the tissue maceration that accompanied insect feeding resembled that resulting from the incubation of excised salivary glands with plant tissues, concluded that the principal damage caused by lygus feeding was due to the action of this salivary PG. Shackel et al. [10] simulated lygus feeding damage in alfalfa and cotton flowers using small glass capillaries of an overall size and shape similar to that of lygus stylets and found that plant damage symptoms caused by the injection of crude and partially purified PG protein solutions from lygus heads and isolated salivary glands were similar to those caused by lygus feeding. On the other hand, injection with the same volume of a solution without PG did not generate any symptoms. Celorio-Mancera et al. [11] compared the effects of a wild type enzymatically active PG and an inactive mutant PG on alfalfa florets using a similar micro-injection method and demonstrated that the enzymatic activity rather than the PG protein structure per se elicited damage symptoms. To date, multiple forms of PG proteins have been biochemically identified in lygus saliva using thermal stability analysis [12], polyacrylamide gel electrophoresis (PAGE) [10], and high-performance anion-exchange chromatography with pulsed amperometric detection (HPAE-PAD) [13]. Recently, the presence of PG genes in lygus was demonstrated using molecular methods [11,14,15], and their expression patterns and functions have been further studied [15–16].

The roles of PG enzymes in plant pectin breakdown, which allows for the better uptake of plant tissue and possibly oviposition site preparation, have also been determined in many phytophagous mirid bugs from other genera in Miridae [17,18]. These previous findings suggest that PG proteins may play a key role in *A*. *lucorum*, favoring plant feeding and eliciting plant injury. Recently, a micro-injection trial showed that partially purified PG proteins of *A*. *lucorum* elicited the same damage symptoms in cotton plants as direct feeding, which further supports our expectation (Lu Yanhui, unpublished data). To explore the PG gene family of *A*. *lucorum*, we constructed a full-length cDNA library from salivary glands, successfully identified 14 PG genes, and investigated the expression pattern of these PG genes in different tissues and at different developmental stages.

## Materials and Methods

### Ethics Statement

No permit was required to collect the tested insects. Sampling did not involve regulated, endangered or protected species.

### Insects

*A*. *lucorum* nymphs and adults were collected from a cotton field at the Langfang Experimental station of the Chinese Academy of Agricultural Sciences, Hebei Province (39.53°N, 116.70°E), China. A laboratory colony was established and maintained at 29 ± 1°C and 60 ± 5% RH, with a 14:10 h (L:D) photoperiod, in climate-controlled rearing chambers and reared on green bean pods (*Phaseolus vulgaris* L.) and a 10% sucrose solution [19].

A cDNA library was created using the salivary glands from 5-day-old adults (500 of each sex). The tissues used for PG transcript expression profiles were also collected from 5-day-old male and female adults. The tissues used were the salivary gland, head (without salivary gland), thorax, abdomen, wing and leg. PG gene expression was also investigated at different stages, including nymphs (1^st^, 3^rd^ and 5^th^ instar) and male and female adults (1, 5, 20 and 30 days post-adult emergence). Three biological replicate groups for each treatment were assayed as triplicate technical replicates. Each group contained thirty eggs, ten nymphs or ten adults of each sex.

### RNA extraction and first-strand cDNA synthesis

Total RNA was isolated using Trizol reagent (Invitrogen, Carlsbad, CA, USA) following the manufacturer’s instructions. The integrity of the total RNA was examined using 1.1% agarose electrophoresis, and the purity was determined by the ratio of A260/A280, as measured by a spectrophotometer. Samples with OD values between 1.9 and 2.0 were used. The first strand cDNA was synthesized using oligo (dT) and M-MLV Reverse Transcriptase.

### Construction of the salivary gland cDNA library

A Creator SMART cDNA Library Construction Kit was used for the construction of the cDNA library according to the manufacturer’s instructions. Briefly, total RNA (1 μg) from salivary glands was used with 1 μl of SMART IV oligonucleotide and 1 μl of CDSIII/30-PCR primer for first-strand cDNA synthesis. The first-strand cDNA was initially amplified by long-distance PCR (LD-PCR) (Advantage 2 Polymerase Mix) using hotstart amplification at 72°C for 10 min, 95°C for 1 min followed by 26 cycles at 95°C for 15 sec and 68°C for 8 min to make double-strand cDNA. The cDNA was then treated with proteinase K at 45°C for 20 min to inactivate DNA polymerase. After proteinase K treatment, the cDNA was digested with *Sfi* I. Size fractionation of the double strand cDNA was performed with a CHROMA SPIN-400 Column. The size distribution of the resulting double-stranded cDNA was visualized by electrophoresis on a 1.1% agarose gel, and fractions containing fragments above 500 were selected, pooled, ethanol-precipitated and ligated to the modified pDNR-LIB plasmid using the asymmetric Sfi I sites. The ligated vector/cDNA mixture was electroporated into ElectroMAX DH5a-E cells. The titer of the primary library was calculated by the dilution titration of bacterial cells onto chloramphenicol plates. The recombination efficiency and average insert size were evaluated by PCR analysis of more than 1000 randomly selected library clones. To make a large, stable quantity of a high-titer stock of the library, the primary cDNA library was amplified.

Prior to sequence analysis, low quality sequences (60 of 5133) and vector contaminated samples were omitted. The remaining high quality ESTs (5073) were assembled into contigs using PHRAP (Phil Green; http://www.phrap.org/phredphrap/phrap.html). The unique assembled contigs were then annotated using BLASTx and BLASTn against the NCBI non-redunant database with an E-value cut off of <10^–5^.

### Sequence analysis of PG genes

Nucleotide acid sequences showed that high identities to PG genes were obtained from the cDNA library. Genes with incomplete coding sequences (CDS) were amplified with specific primers (S1 Table) using a RACE kit (GeneRacer^TM^ Kit) following the manufacturer’s instructions. Gene-specific primers were then designed to amplify the full length cDNA (S1 Table). The cycling conditions were as follows: initial denaturation at 95°C for 3 min, then 33 cycles of 94°C for 1 min, 50°C for 1 min, 72°C for 3 min, and a final extension at 72°C for 10 min.

The percent identities of the amino acid sequences of *A*. *lucorum* PGs obtained in this study and at NCBI were calculated using CLUSTAL W [20]. A neighbor-joining (NJ) tree using Poisson correction distances and bootstrap values (1000 replicates) with the amino acid sequences of the PGs was constructed using CLUSTAL W and MEGA5.0 [21].

### Expression of PG genes

Total RNA was isolated using the whole body of *A*. *lucorum*, and cDNA was synthesized from each individual adult as described above. Using *β-actin* (GenBank accession numbers: JN616391) and *GAPDH* (GenBank accession numbers: JX987672) as the reference genes, real-time PCR (qPCR) was carried out following the TaqMan method in a 20-μl reaction composed of 1 μl of template cDNA, 2×Maxima^TM^ Probe/ROX qPCR Master Mix, 0.3 μM of each primer and 0.2 μM probe (S1 Table) in a 7500 Fast Real-time PCR System. The thermal cycling conditions were 50°C for 2 min, 95°C for 10 min followed by 45 cycles at 95°C for 15 s and 60°C for 60 s. Fluorescence data were collected at the end of each cycle. Each sample was run in triplicate. No-template negative controls were included. The fold differences of the genes were calculated according to the 2^-△△CT^ method [22]. Three repeats were used for each data-point.

To compare the proportional changes of the PG mRNA levels in the whole body, the absolute quantification methodology using a standard curve was adopted [23]. Fragments containing the primers and probes from the qPCR of the PG genes were amplified with our de novo primers (S1 Table) and cloned into the pEASY-T Cloning Vector. These plasmids were serially diluted 1:10 to generate the standard curve, and six concentrations and three technical replicates were included.

Statistical analyses were conducted using STATA v.9.0. Student’s t-test or ANOVA with Bonferroni multiple comparisons were used to determine the level of significance in the relative levels of mRNA expression.

## Results

### Sequence analysis of ESTs from a salivary gland cDNA library

A normalized salivary gland cDNA library was constructed for *A*. *lucorum* via the DSN normalization method combined with the SMART technique according to the manufacturer’s instructions. The titer of the library was approximately 1.7 × 10^7^ pfu/ml, indicating an adequate representation of the expressed genes. We selected 5073 ESTs from the library for further analysis. The majority of ESTs ranged from 500 bp to 1300 bp with 99% recombinant efficiency, and the mean length was 1104 bp for ESTs from the salivary gland library (S1 Fig). The 5073 ESTs from *A*. *lucorum* salivary glands produced 3642 unigenes. BLASTx analysis facilitated the annotation of all 3642 unigenes, 77 of which (S2 Table) had high sequence identities with PG genes from other species of mirid bugs.

### Sequence alignment and phylogenetic analysis of the PGs

These 77 PG-like contigs and unigenes represent 14 PG transcripts (Genbank accession numbers: KF881899~KF881912), and these transcripts were confirmed by PCR with specific primers (S1 Table). The *A*. *lucorum* PG transcripts, including the ones reported in this study and those obtained from NCBI, had amino acid sequence identities of 30.3–98.9%, in which PG1-3, PG2-1, PG2-2, PG3-5 and PG5-1 showed identities more than 95% with those obtained from NCBI (S3 Table). All sequences contained conserved PG motifs, including the predicted N-terminal signal peptides, putative disulfide bridges and enzymatically critical amino acid motifs (S2 Fig).

**Fig 1:**
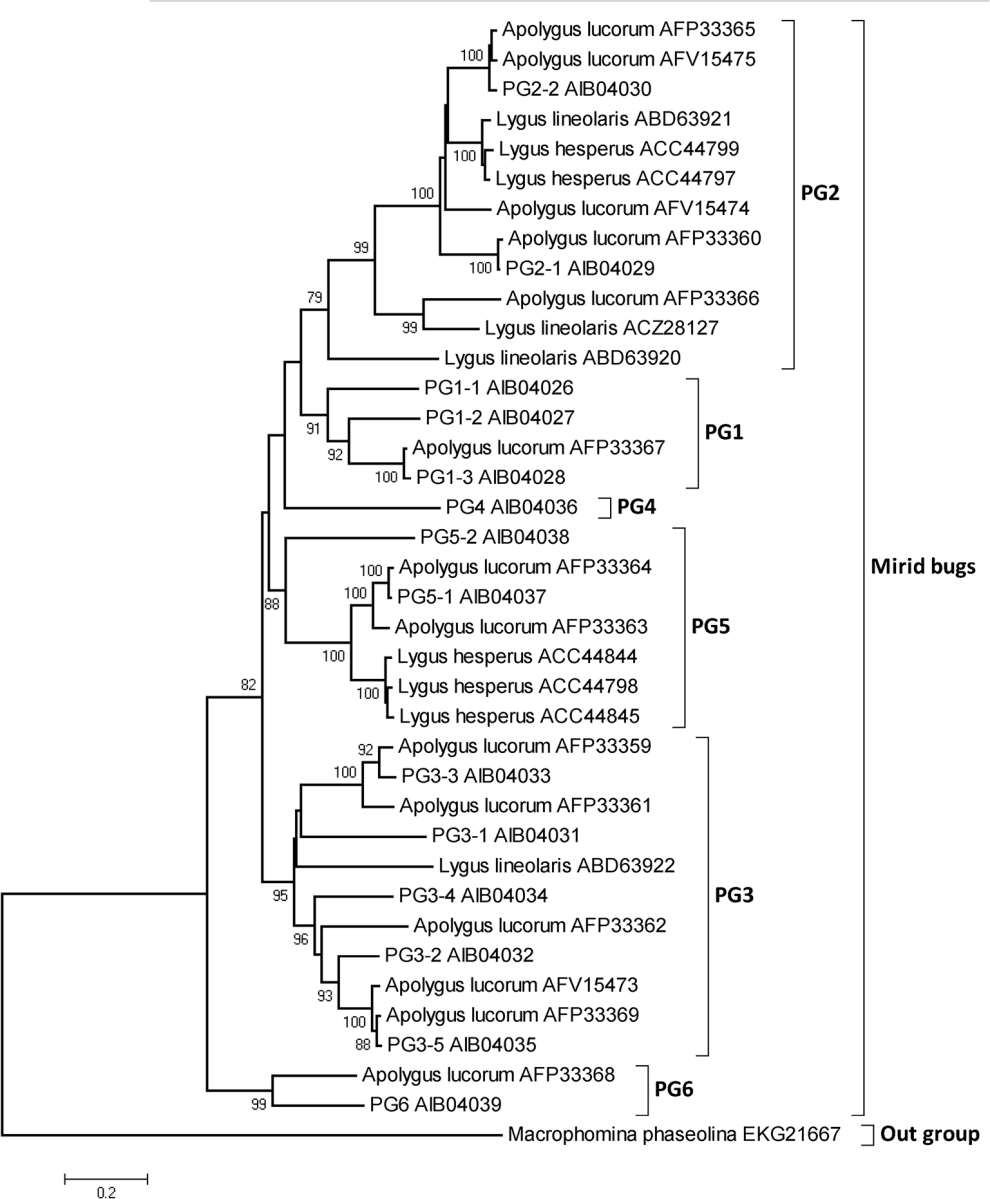
Phylogenetic analysis of PG transcritps. A neighbor-joining tree using Poisson-correction distances was constructed with PG amino acid sequences from three mirid bugs, with a glycoside hydrolase from *Macrophomina phaseolina* MS6 as an outgroup. Bootstrap values (1000 replicates) are indicated on the nodes, and those lower than 75 are not shown. The abbreviations represent the PGs from *Apolygus lucorum* identified in this study. PGs identified previously are indicated by the full *Apolygus lucorum* name and the associated accession number.

A neighbor-joining tree was constructed using the deduced amino acid sequences of mirid bugs, with a glycoside hydrolase from a Botryosphaeriaceae fungus, *Macrophomina phaseolina* (Tassi) Goid, as the outgroup (Fig 1). Taking the identities, phylogenetic analysis results and names of the PGs reported in other mirid bugs into account, we divided the mirid bug PGs into six clusters, named PG1-PG6 (Table 1).

**Table 1:** Bioinformatic analysis of *Apolygus lucorum* PGs.

Name of PGs	GenBank accession No.	Length of ORF (bp)	Number of amino acid (aa)	Molecular weight (kDa)	Isoelectricpoint
PG1-1	KF881899	1068	355	38.62	10.01
PG1-2	KF881900	1071	356	38.53	9.61
PG1-3	KF881901	1071	356	38.54	9.92
PG2-1	KF881902	1098	365	40.25	9.79
PG2-2	KF881903	1101	366	40.08	9.28
PG3-1	KF881904	1092	363	39.3	8.47
PG3-2	KF881905	1047	348	37.75	10.19
PG3-3	KF881906	1071	356	38.99	8.48
PG3-4	KF881907	1050	349	37.99	9.42
PG3-5	KF881908	1053	350	37.72	9.33
PG4	KF881909	1071	356	38.52	10.21
PG5-1	KF881910	1041	346	36.96	10.39
PG5-2	KF881911	1077	358	38.56	9.44
PG6	KF881912	106	355	38.68	9.04

### Expression patterns of PG genes in different tissues

**Fig 2:**
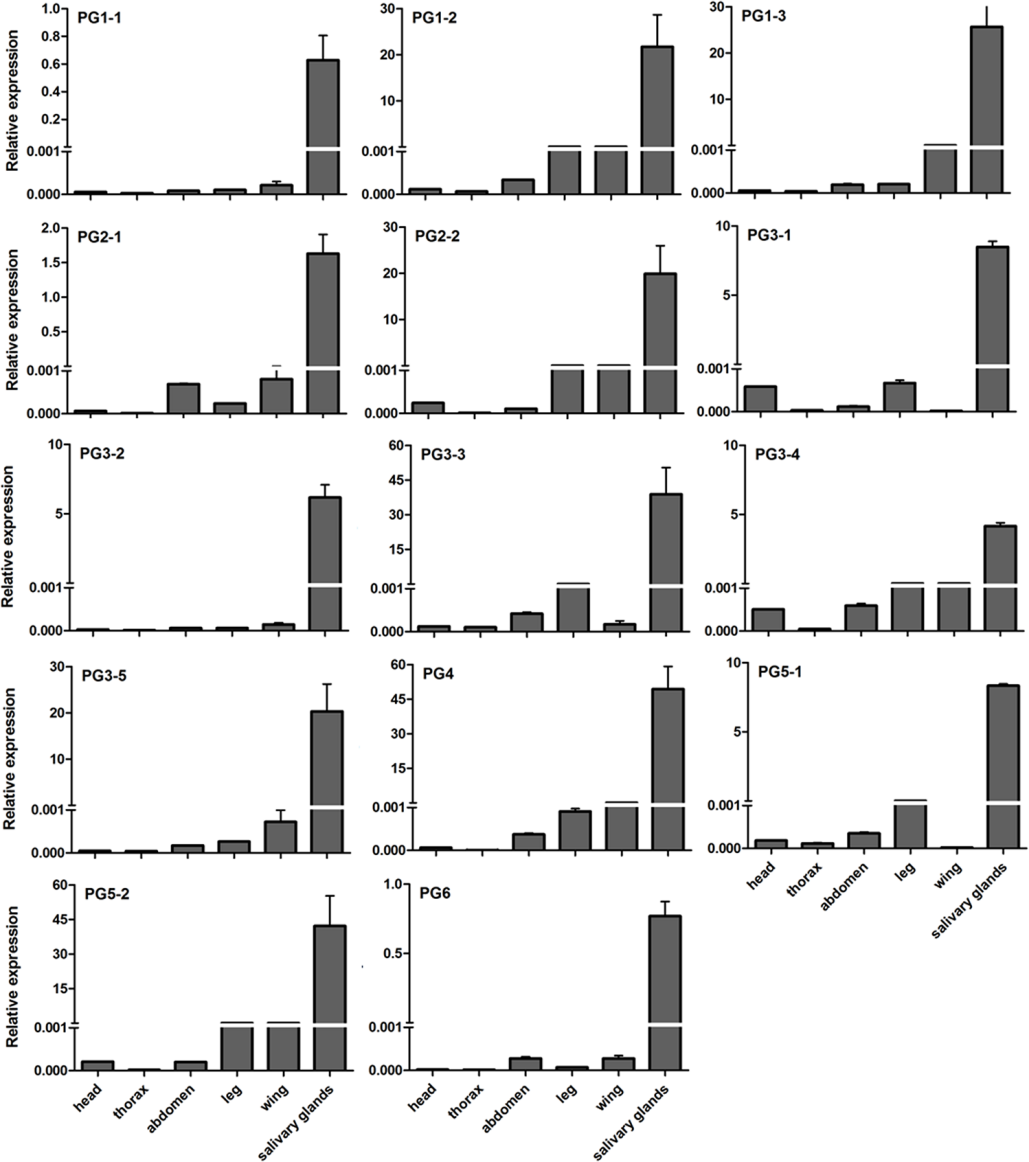
Relative expression level of PG transcripts in different tissues from *Apolygus lucorum* adults using *β-actin* and GAPDH as reference genes. The values on the Y-axis indicate the expression level of PG genes relative to *β-actin* and GAPDH genes, which is set to 1. Mean ± SE. The expression level of each PG was significantly higher in salivary glands than in other tissues (P<0.05).

To determine the level of each PG transcript from *A*. *lucorum*, primers and TaqMan-probes were designed according to the specific regions. Using *β-actin* and GAPDH as reference genes, the tissue distribution showed that the expression levels of the 14 PGs were significantly higher in salivary glands than in other tissues, including the head, thorax, abdomen, leg and wing (P<0.05) (Fig 2).

### Expression profiles of PG transcripts at different developmental stages

**Fig 3:**
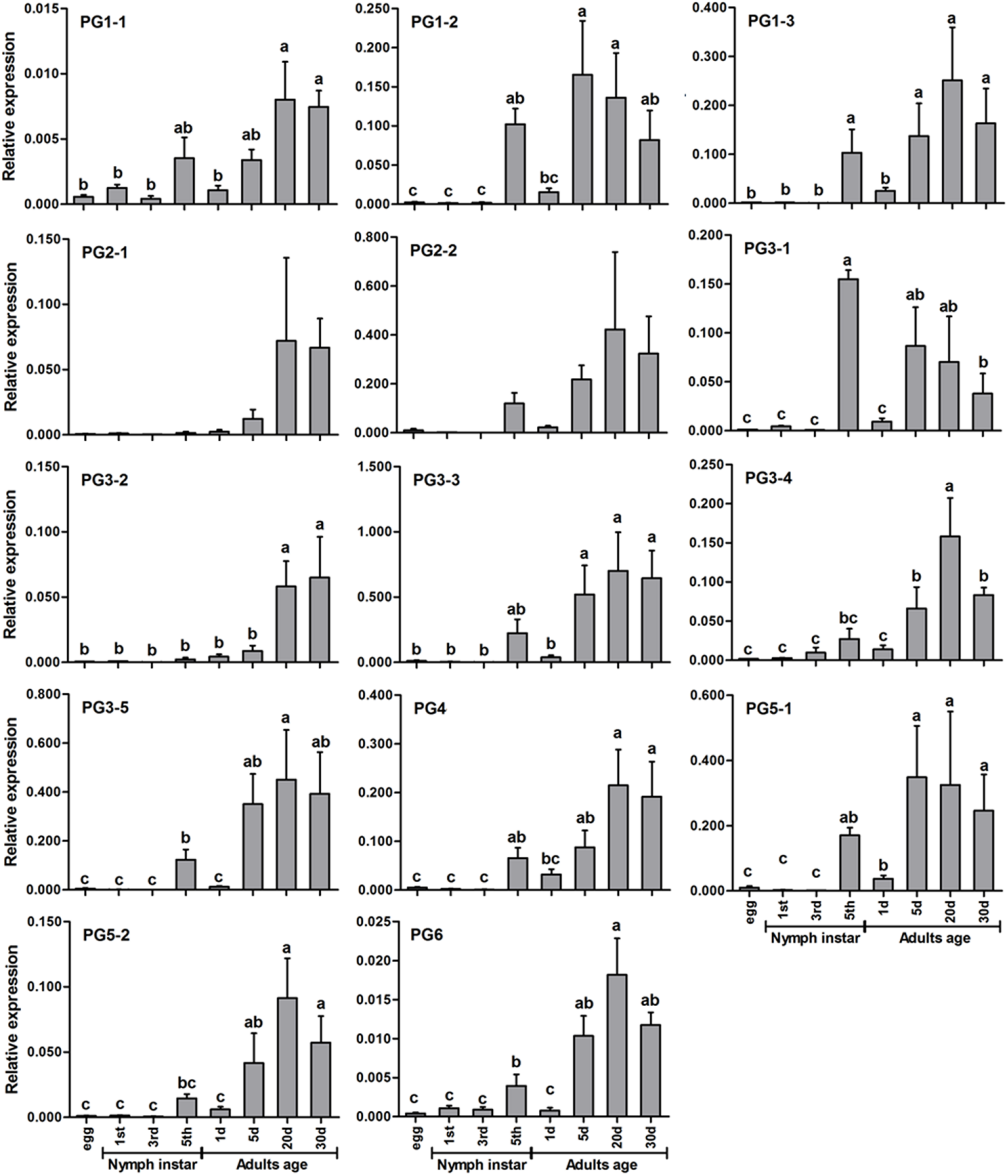
Relative expression levels of PG transcripts with *β-actin* and GAPDH as reference genes in *Apolygus lucorum* at different developmental stages. The values on the Y-axis indicate the expression level of PG genes relative to *β-actin* and GAPDH genes, which is set to 1. Mean± SE. Significant differences (P<0.05) are indicated with different letters.

The qPCR analysis using the average Ct values of *β-actin* and GAPDH revealed significant fluctuation in all 14 PGs (Fig 3). The expression levels of all of the genes in egg and 1^st^ and 3^rd^ instar nymphs were very low, and those in 5^th^ instar nymph were greatly increased. The ANOVA results for all developmental stages showed that there were significant differences between the egg and nymphal stages for PG1-2 and PG3-1 (P<0.05), but not for other PG genes (P>0.05). The PG expression levels of 5^th^ instar nymphs and 1-day-old adults (i.e., shortly before and after adult emergence) usually did not change (P>0.05), and a significant difference was only found for PG3-1 (P<0.05). There was a general trend of increasing PG expression between 5 and 20-day-old adults. Significant differences (P<0.05) were found between the 1, 5 and 20-day-old adults for 12 PGs (PG1-1, PG1-2, PG1-3, PG2-1, PG2-2, PG3-2, PG3-3, PG3-4, PG3-5, PG4, PG5-2, and PG6). No significant differences among the PG transcripts (P>0.05) were found between the 20 and 30-day-old adults. Additionally, the expression levels of PGs at various adult stages were not significantly different between males and females, except for the 5-day-old adults, at which point the levels in females were significantly higher than those in males (S4 Table).

**Fig 4:**
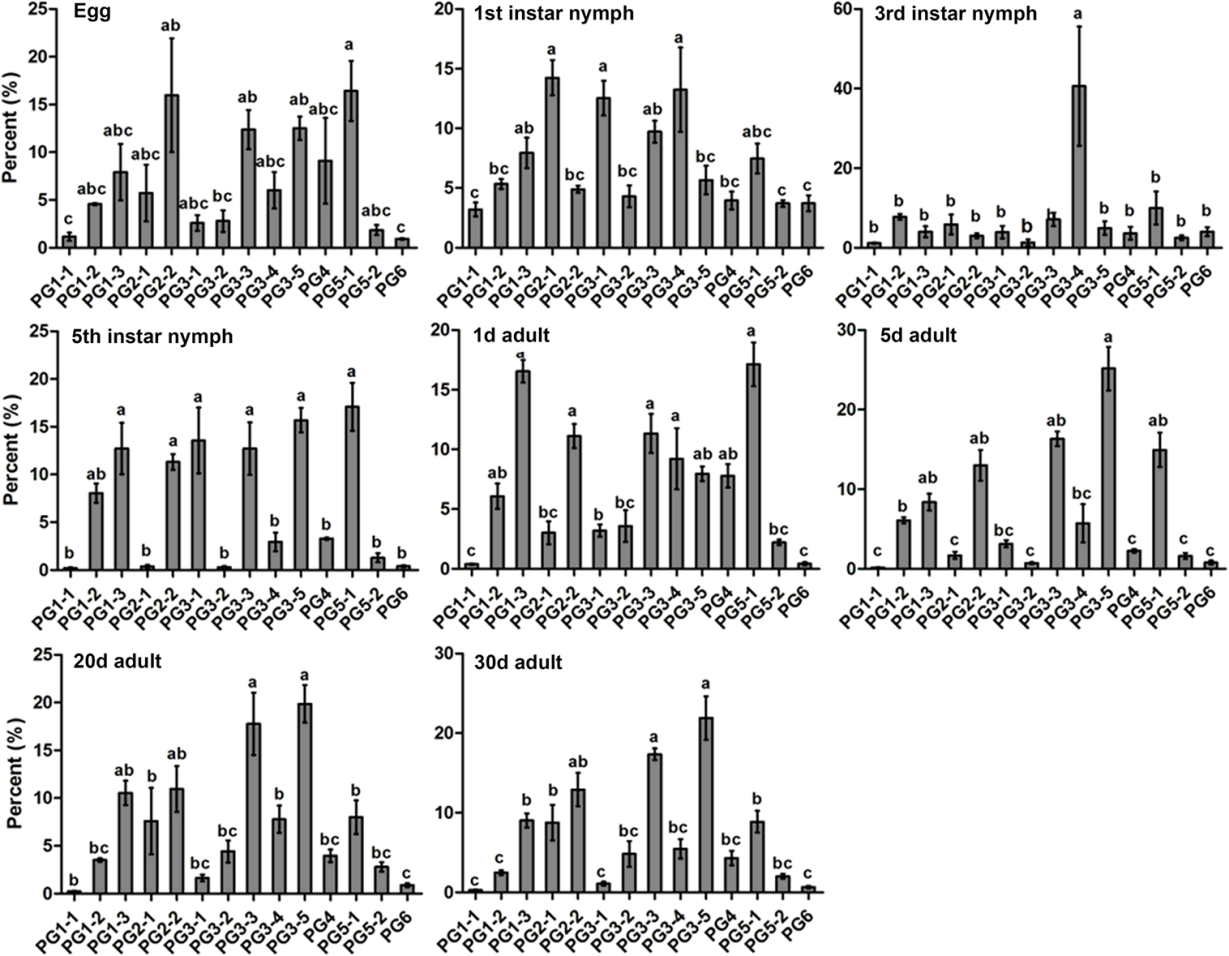
Proportional expression of PG genes in *Apolygus lucorum* at different stages. Mean ± SE. Significant differences (P<0.05) are indicated with different letters.

Standard curves for each PG gene were constructed (S3 Fig) and served as proportional measures of PG expression at different stages. At the nymphal stages, six of the 14 transcripts, including PG1-1, PG2-1, PG3-2, PG4, PG5-2, and PG6, always had low proportional levels of expression. Interestingly, PG3-4 was proportionally expressed at a significantly higher level than other transcripts at 3^rd^ instar nymph. At the adult stages, six transcripts, including PG1-1, PG2-1, PG3-1, PG3-2, PG5-2, and PG6, were always expressed at low levels (Fig 4).

## Discussion

The function of PG proteins in mirid bugs has been widely studied, and to date, the complete cDNA sequences of several PGs have been cloned from *Lygus lineolaris* using Expressed Sequenced Tags [14]. In this study, we cloned PGs by constructing a cDNA library in *A*. *lucorum*, an important crop pest in China [1,4], and determined their expression pattern in different tissues and developmental stages of this mirid bug.

Using BLASTx and BLASTn, we identified 14 PG genes from 5073 ESTs of the *A*. *lucorum* cDNA library. The amino acid sequences of these PGs are highly similar to the PGs of two other mirid bugs (*Lygus hesperus* and *Lygus lineolaris*) and those previously reported in *A*. *lucorum* with respect to the predicted signal peptide, molecular weight and conserved domains [15,16]. PG nomenclature for mirid bugs is difficult due to the limited amount of information that is available. The neighbor-joining tree based on amino acid sequences clustered the PGs into six main clades, suggesting that gene duplication occurred many times in mirid bugs, and according to the previous name of PGs in mirid bugs [13, 15], the clades were named PG1-PG6. With the exception of PG6, the PGs clustered into two branches: one contained PG1, PG2 and PG4, and the other included PG3 and PG5. PGs typically have endo- and exo-activity [13], but the activity of the various PGs of *A*. *lucorum* should be further investigated. In this study, 14 PGs from *A*. *lucorum* were reported and 9 of these sequences showed identities lower than 95% and were substantially different from the *A*. *lucorum* PGs obtained from NCBI. Of the 14 PGs reported in this study, five were more than 95% identical with the sequences obtained from NCBI. These differences may reflect the disparate populations of *A*. *lucorum* used in the present study and other studies. These results suggested there were at least 21 PGs in *A*. *lucorum* divided into 6 groups.

PGs are predominantly expressed in the salivary glands of *L*. *lineolaris* [15,16]. Our results also showed that the 14 PGs identified in this study were also highly expressed in the salivary glands of *A*. *lucorum*. These PGs exhibited different expression patterns during different developmental stages. In general, the older nymphs and adults of *A*. *lucorum* expressed high levels of PGs, which was also observed in *L*. *lineolaris* [15]. Late instar nymphs of *A*. *lucorum* usually cause greater damage to plant leaves than adults [2]; however, PG expression levels are generally greater at the adult stages. This inconsistency may result from differences in their feeding habits. More specifically, the nymphs feed more on food plants for rapid development, while the adults mainly prefer to feed on flowers and nectars [24,25]. Additionally, the adults are more mobile and can feed on diverse host plants, whereas nymphs stay on only one host plant.

For single copy genes, the assessment of gene-expression relative to the housekeeping genes is usually available by selecting suitable development stages for RNAi studies. However, for multigene families, selecting dominantly expressed genes and suitable developmental stages for RNAi studies is not always so straightforward [26]. The proportional expression analysis indicated that several PGs (e.g., PG1-1, PG2-1, PG3-2, PG5-2, and PG6) of *A*. *lucorum* were continuously expressed at low levels throughout all of the detected stages, and the expression levels of other PGs identified in this study usually changed according to the developmental stage. PG3-4 was the predominant PG during both the 3^rd^ and 4^th^ instar stages, suggesting that this PG may play a dominant role throughout this developmental period. Micro-injection [10] and manual injection [8] have been developed to simulate mirid bug feeding on host plants under laboratory and field conditions. The role of different PGs (recombinant proteins produced by prokaryotic and eukaryotic cells) in plant feeding and injury caused by *A*. *lucorum* in different plant species and tissues could be further evaluated using the above injection methods.

In conclusion, the mirid bug PGs are a multigene family. We used PCR methods to identify 14 PG sequences from an *A*. *lucorum* salivary gland cDNA library. The PGs were predominantly expressed in salivary glands and highly expressed in 5^th^ instar stages of nymphs and older adults. The results provide an important basis for evaluating the role of different PGs in eliciting plant injury caused by this economically significant mirid bug, as well as for understanding the interactions between *A*. *lucorum* and its host plants [18].

## Supporting Information

S1 Fig Distribution of the sequence length of the unigenes in the salivary gland of *Apolygus lucorum* cDNA library. (PDF)

S2 Fig Alignments of the amino acid sequences of PGs from mirid bugs. (PDF)

S3 Fig Standard curves for PG genes of *Apolygus lucorum* determined by triplicate sampling. (PDF)

S1 Table Primers used for PG genes. (PDF)

S2 Table Sequences from the cDNA library showing high identities with PG genes. (PDF)

S3 Table Identities of the PG amino acid sequences from *Apolygus lucorum*. (PDF)

S4 Table Paired Student's t-test of relative PG mRNA expression levels between male and female adults of *Apolygus lucorum*. (PDF)
