# Supplementary material for: Molecular Characterization and Expression Profiles of Polygalacturonase Genes in Apolygus lucorum (Hemiptera: Miridae)
Source: PLoS One. 2015 May 8;10(5):e0126391. doi: 10.1371/journal.pone.0126391 (PMC4425681; doi:10.1371/journal.pone.0126391)
Supplement: S1 Fig — (PDF) [file pone.0126391.s001.pdf]

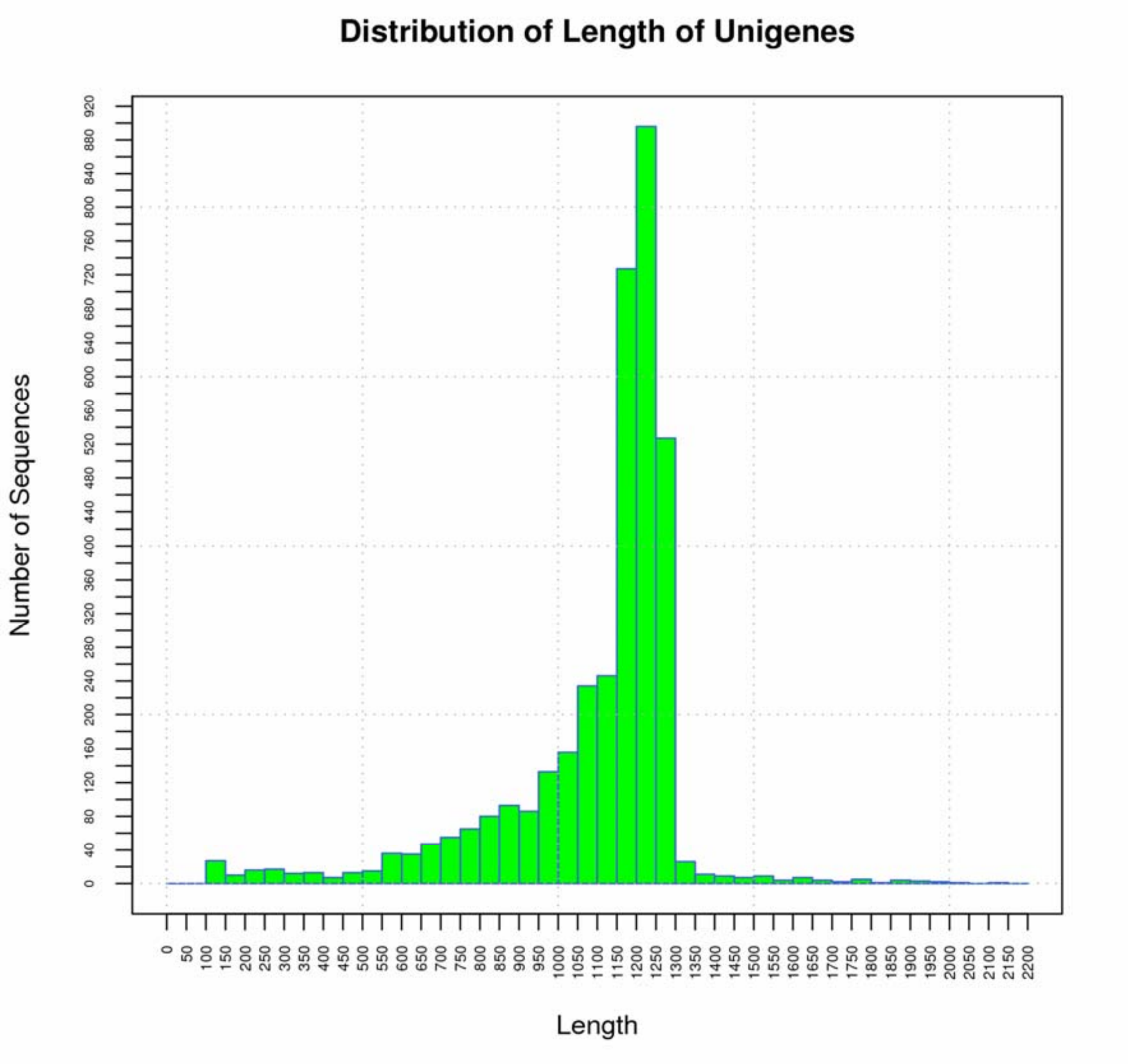

S1 Fig. Distribution of the sequence length of the unigenes in the salivary gland of *Apolygus lucorum* cDNA library
