# Supplementary material for: Molecular Characterization and Expression Profiles of Polygalacturonase Genes in Apolygus lucorum (Hemiptera: Miridae)
Source: PLoS One. 2015 May 8;10(5):e0126391. doi: 10.1371/journal.pone.0126391 (PMC4425681; doi:10.1371/journal.pone.0126391)
Supplement: S2 Fig — (PDF) [file pone.0126391.s002.pdf]

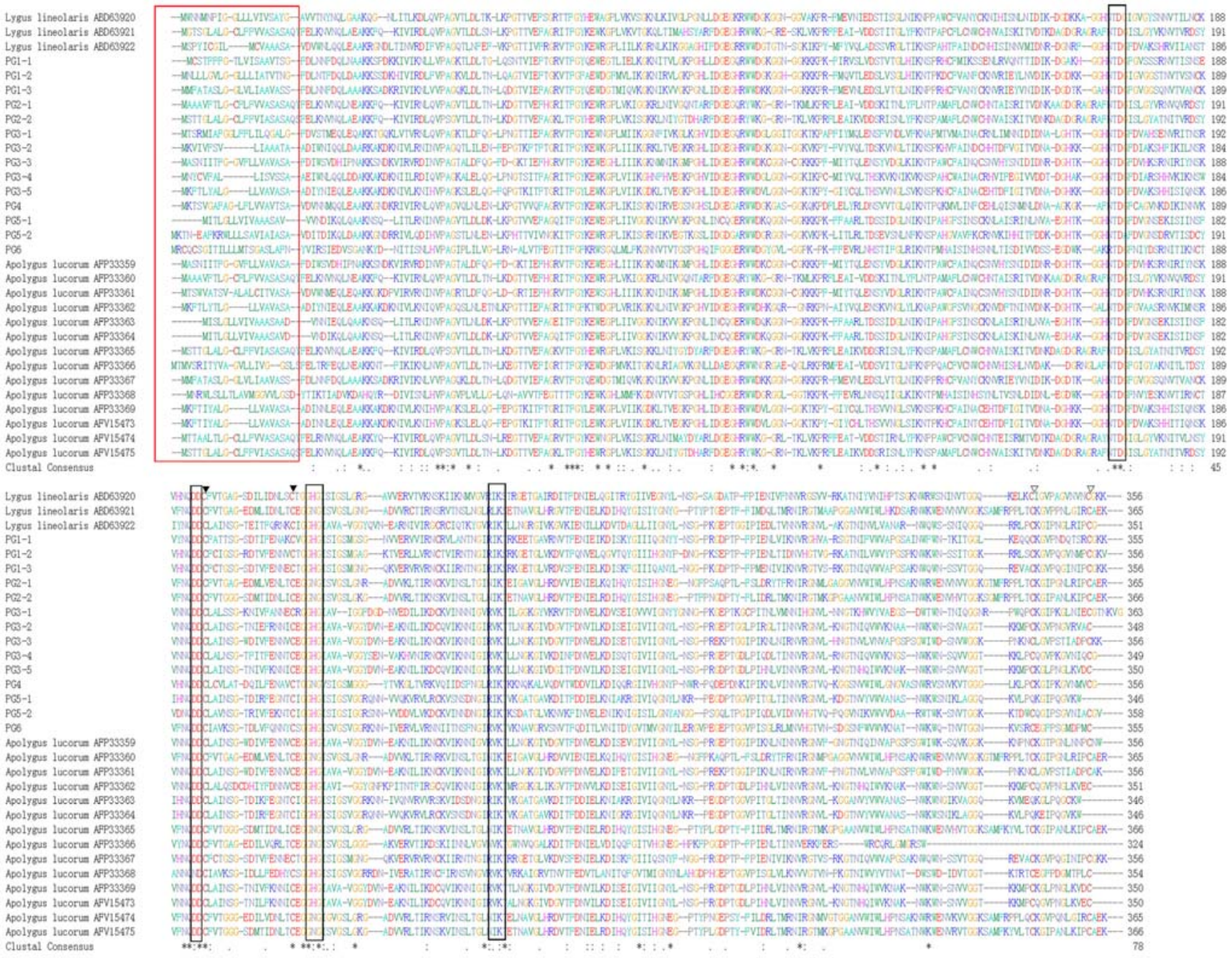

S2 Fig. Alignments of the amino acid sequences of PGs from mirid bugs. The red line box represents predicted N-terminal signal peptides. The black boxes stand for enzymatically critical amino acid motifs. ▼ and ▽ represent putative disulfide bridges. - = gaps. * = identical residues, : = strong positive residues, . = weaker positive residues.
