# Supplementary figures and images for: Molecular Characterization and Expression Profiles of Polygalacturonase Genes in Apolygus lucorum (Hemiptera: Miridae)
Source: PLoS One. 2015 May 8;10(5):e0126391. doi: 10.1371/journal.pone.0126391 (PMC4425681; doi:10.1371/journal.pone.0126391)

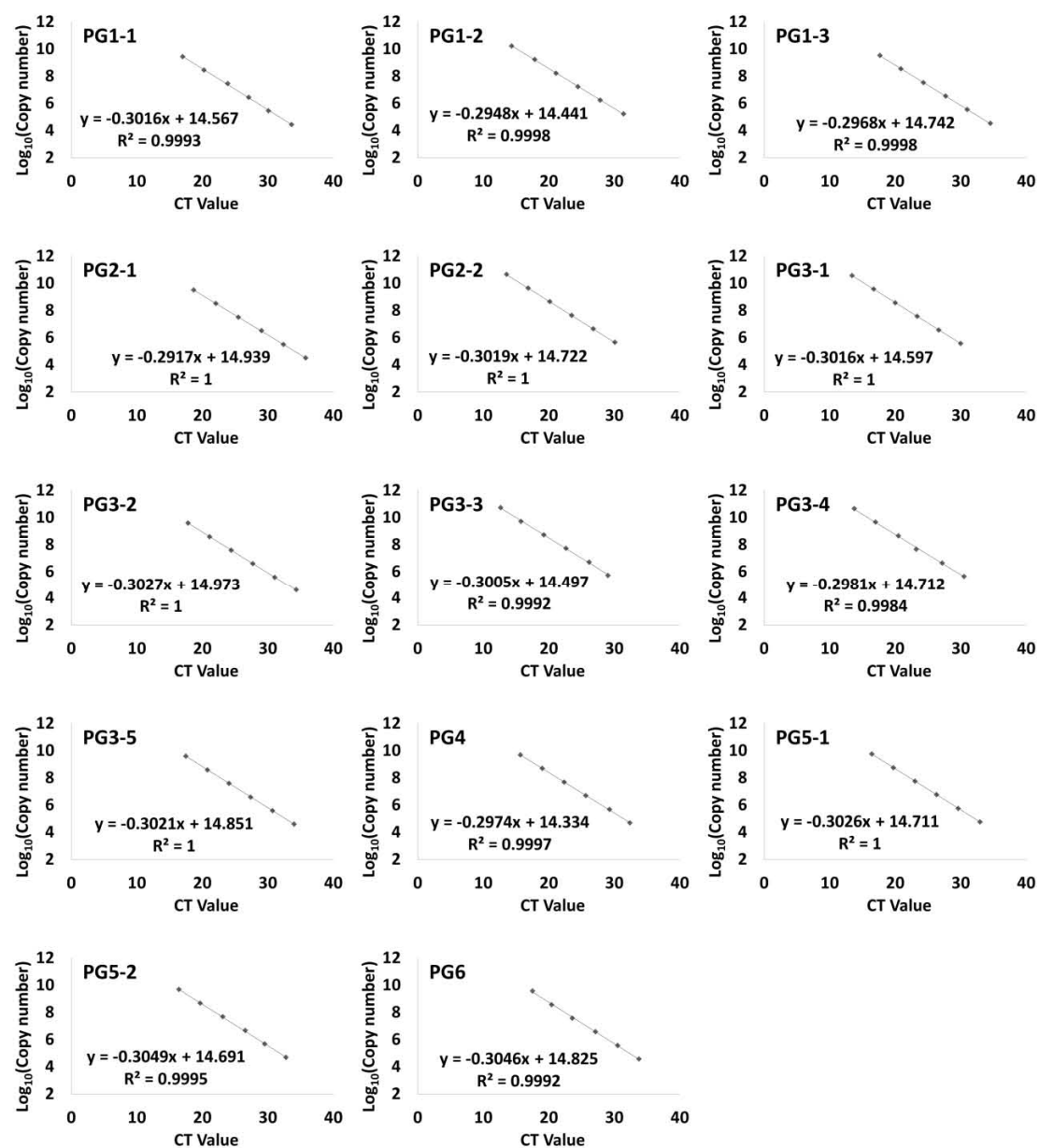

**S3 Fig.** Standard curves for PG genes of *Apolygus lucorum* determined by triplicate sampling.

Supplement: S3 Fig — (PDF) [file pone.0126391.s003.pdf]
