# Supplementary material for: Molecular Characterization and Expression Profiles of Polygalacturonase Genes in Apolygus lucorum (Hemiptera: Miridae)
Source: PLoS One. 2015 May 8;10(5):e0126391. doi: 10.1371/journal.pone.0126391 (PMC4425681; doi:10.1371/journal.pone.0126391)
Supplement: S1 Table — (PDF) [file pone.0126391.s004.pdf]

S1 Table. Primers used for PG genes

Primer name	Sequence (5'-3')	Type
PG1-1F-3RACE	GAACACTAAAATCACTGGCGG	3’RACE and 5’RACE for PGs from *Apolygus lucorum*
PG1-2F-3RACE	GTGAACCCACTCCATTCCCCATCGAA	
PG1-3F-3RACE	GAGAGACTGGTTTGGTTAGGGATGTA	
PG2-1F-3RACE	GACGACTGCTTTGTCACTGGAGGTGG	
PG2-2F-3RACE	AACATCAAGACGGAAACCAACGCA	
PG3-1F-3RACE	CAAGGACGTCAGCGAAATTGGTGT	
PG3-2F-3RACE	ACACTGAACGGCAAAGGTATCG	
PG3-3F-3RACE	AGGGAGGTCACGGTATCGCTGTCG	
PG3-4F-3RACE	CGTGCTCAGAAACGGAACCAACAT	
PG3-5F-3RACE	TCGCTGTTGCTGTAGGAGGTTATG	
PG4 F-3RACE	GAACTGGAGAGTCAGCAACGTGAA	
PG5-1F-3RACE	CAAGCGTCCTGAAGGCGACCCAAC	
PG5-2F-3RACE	CAAAGTTTGGGTGGTCGATGCCGC	
PG6 F-3RACE	TAGGTAACTACATCCTGGAGCGAG	
PG1-1R-5RACE	TTTGAAATGAAATCAGAGCCT	
PG1-2R-5RACE	TACGAACAGGTCCCGAATAAC	
PG1-3R-5RACE	TTGTCCCACCAACGATGTCCT	
PG2-1R-5RACE	AACAGGAAACATCCAAGGGTG	
PG2-2R-5RACE	CACAAAGAACAAGCACCCAAG	
PG3-1R-5RACE	GTCCAGAGTCTTCCCAGCAGG	
PG3-2R-5RACE	TGCGACCTTTGATGATAATGAG	
PG3-3R-5RACE	CAGCGACGAGAAGGAAGACAC	
PG3-4R-5RACE	TGCCTCCGAGTCCGTCCCACC	
PG3-5R-5RACE	CAATCAAGTGTCCAGGTTTTCC	
PG4 R-5RACE	GGGCTCCTACGCTCGTCTTCA	
PG5-1R-5RACE	GCAGGAGTTGATTGAAAACCCGTG	
PG5-2R-5RACE	TTATCGTCAAATGTGATGTGGTGG	
PG6 R-5RACE	TCTGGGGAGGTGTTCCTAAGC	
PG1-1F	GCAGTGATCGGTTTCGACTGAG	Gene specific primers used for the cloning of the open reading frames of *Apolygus lucorum* PG genes
PG1-1R	GGGAATTAGGGACAATGTGTAT	
PG1-2F	GTGCCGTGTGTTGTTGATGAC	
PG1-2R	TCACGGCATAGTATTTATTCA	
PG1-3F	ACTGCGAGAATGATGTTCGC	
PG1-3R	AGGTACGAGATAGGATTATA	
PG2-1F	TCGAGCAGCACTGAAAGCTA	
PG2-1R	CATTATTCATATTTTATTGC	
PG2-2F	AATTTCTCTCGTTAGCTGCAC	
PG2-2R	ATCTTTTTTTTCTTTATTGAA	
PG3-1F	AGCGCATCTGATTTCAGCTAA	
PG3-1R	AATTGAACAATTCTCTGTATT	
PG3-2F	AGTCTTCGGCAGGCACCAGAAAGC	
PG3-2R	GATAATTGATATATTTTAATTGTC	
PG3-3F	GAGGTGAGATAACTGATTGTG	

PG3-3R	AAACAAGACGTTATCTAACTT	
PG3-4F	TCAAGATCTCAGAGAGAGCGG	
PG3-4R	GACACTTCGAAACACGAGTTT	
PG3-5F	AATATTCTTGCCAACGATTGAACC	
PG3-5R	GTAATGTAATTTTAGTTTATTTCA	
PG4F	CAGTCCCGATTTCTCACAGTT	
PG4R	ACGTTGCGACTAAATTATTTA	
PG5-1F	ACACTTCACCTAGAACAACCTCAAC	
PG5-1R	GCAGTAGATTAGTTTATTGAGTTCC	
PG5-2F	AGACTTTTCATCTCACCAAGA	
PG5-2R	CATAAAATATTTATTTGAGGG	
PG6F	CGAGCTGTCAGCTTAGGAAC	
PG6R	CAAATCCCAACATTCAGTAT	
PG1-1 F	CCAAGCATGGTGGACATAACAC	Oligonucleotide primers used for gene expression analysis of the PG genes from *Apolygus lucorum*.
PG1-1 R	CTCCGACACACTTGGCATTTTC	
PG1-1 Probe	FAM-TACAATCAAGACGACTGCTTTGCGACAACA-TAMRA	
PG1-2 F	TTGATTGACGGTGAAGGCCATA	
PG1-2 R	ATTGGCGACGAAACAATCTTTT	
PG1-2 Probe	FAM-CCACGGAGGCAAAAAGAAGCCCA-TAMRA	
PG1-3 F	TCTAATTGCGGCAGTCGC	
PG1-3 R	CCGCTTGTCAGCACTTTTCTT	
PG1-3 Probe	FAM-CAATAACTTCGACCAACTCGCTGCGG-TAMRA	
PG2-1 F	GGTGCCACAACACTGCTATTTC	
PG2-1 R	TCTTCCAGCCCTGCCGTCT	
PG2-1 Probe	FAM-AGAATAACCGTTGACAACAAAGCTGCCG-TAMRA	
PG2-2 F	CGATCTCCAAAATAACCGTTGA	
PG2-2 R	CGGTGACGAAGCAGTCATCTT	
PG2-2 Probe	FAM-TTGGGTTATGCCACCAACATTACTGTCCG-TAMRA	
PG3-1 F	GAGGACACAACACTGACGGTTT	
PG3-1 R	TTCCATGTCCTCCACGACACT	
PG3-1 Probe	FAM-CCAAGACGACTGTCTGGCTCTCAGCTC-TAMRA	
PG3-2 F	ACGACTGTCATCACACCGACTT	
PG3-2 R	GGCAGTCGTCTTGATTGTTAACTC	
PG3-2 Probe	FAM-CACAAGAAAGGAGGCCACAACACAGACG-TAMRA	
PG3-3 F	TCAACGTTCCCGCTGGAA	
PG3-3 R	GTAACGCGTCCATGGAATTCTAT	
PG3-3 Probe	FAM-TGCACTTGACTTCCAAGGTTTCGACG-TAMRA	
PG3-4 F	TCATCGGCAACTACCTGAACTC	
PG3-4 R	ATTTCCAGTTGGATCCGTTCTT	
PG3-4 Probe	FAM-CTCCCCATCCAGGATCTGACCATCA-TAMRA	
PG3-5 F	ATGTACCCGCTGGAAAGTCATT	
PG3-5 R	CAGGTTTTCCCTCGACAGTCAA	
PG3-5 Probe	FAM-AACCAAAATTACTTTCACTGGCCGCATCA-TAMRA	
PG4 F	CGTCATTCTCAAGGATATCCAACA	
PG4 R	TTGGAACCTCCCTTTTGAACAG	
PG4 Probe	FAM-AATCATCGTCCACGGAAACTACCCCAAC-TAMRA	
PG5-1 F	ACTTCAAGCGGCCAAAAACTCT	
PG5-1 R	ATCTGCCCAGCGAATTCCA	

PG5-1 Probe	FAM-TCAATGTTCCTGCTGGAGTCACGCTCG-TAMRA
PG5-2 F	AACGTCGTTGATGATGTCCTTGT
PG5-2 R	TCCACCGTTAGCATAGTTACCAA
PG5-2 Probe	FAM-AAGGTAATCAACAACGACAACGGAATCCGC-TAMRA
PG6 F	CATCGCAGTCAAATCAGGAACT
PG6 R	ACCAGCACTCGTTCCACAATAT
PG6 Probe	FAM-CCAGAACAACTACTGCTCGGGAGGACA-TAMRA
Actin F	TCTTGCGGTATCCACGAAAC
Actin R	GAGGGCGGTGATTTCCTTCT
Actin Probe	FAM-ACTCCATCATGAAGTGCGACGTCGA-TAMRA
GAPDH F	CTGGAGCTGAATACGTCGTTGAA
GAPDH R	GAGGGATCGTAGGCTTCCAAGTT
GAPDH Probe	FAM-AAAGCAAAGGCCCACATCGACGGA-TAMRA
