# Supplementary material for: Molecular Characterization and Expression Profiles of Polygalacturonase Genes in Apolygus lucorum (Hemiptera: Miridae)
Source: PLoS One. 2015 May 8;10(5):e0126391. doi: 10.1371/journal.pone.0126391 (PMC4425681; doi:10.1371/journal.pone.0126391)
Supplement: S2 Table — (PDF) [file pone.0126391.s005.pdf]

**S2 Table. Sequences from the cDNA library showing high identities with PG genes.**

| GeneID | BestHits | Identity | E-value | Description |
|---|---|---|---|---|
| Contig560 | gi\|399207673\|gb\|AFP33367.1\| | 66.39 | 1.00E-98 | polygalacturonase 6 [Apolygus lucorum] |
| Contig560 | gi\|145098373\|gb\|ABD63920.1\| | 52.85 | 3.00E-77 | polygalacturonase PG1 [Lygus lineolaris] |
| Contig560 | gi\|409150895\|gb\|AFV15473.1\| | 46.92 | 7.00E-65 | polygalacturonase PG1 [Apolygus lucorum] |
| Contig520 | gi\|399207673\|gb\|AFP33367.1\| | 70.09 | 3.00E-166 | polygalacturonase 6 [Apolygus lucorum] |
| Contig520 | gi\|145098373\|gb\|ABD63920.1\| | 57.58 | 6.00E-133 | polygalacturonase PG1 [Lygus lineolaris] |
| Contig520 | gi\|409150895\|gb\|AFV15473.1\| | 50.57 | 2.00E-101 | polygalacturonase PG1 [Apolygus lucorum] |
| XT5K-10_E04_024 | gi\|399207673\|gb\|AFP33367.1\| | 68.92 | 6.00E-95 | polygalacturonase 6 [Apolygus lucorum] |
| XT5K-10_E04_024 | gi\|399207677\|gb\|AFP33369.1\| | 54.84 | 5.00E-67 | polygalacturonase 9 [Apolygus lucorum] |
| XT5K-10_E04_024 | gi\|409150895\|gb\|AFV15473.1\| | 55.3 | 6.00E-67 | polygalacturonase PG1 [Apolygus lucorum] |
| XT5K-13_E08_056 | gi\|399207673\|gb\|AFP33367.1\| | 68.9 | 4.00E-117 | polygalacturonase 6 [Apolygus lucorum] |
| XT5K-13_E08_056 | gi\|145098373\|gb\|ABD63920.1\| | 53.96 | 2.00E-81 | polygalacturonase PG1 [Lygus lineolaris] |
| XT5K-13_E08_056 | gi\|409150895\|gb\|AFV15473.1\| | 51.77 | 1.00E-78 | polygalacturonase PG1 [Apolygus lucorum] |
| XT5K-14_B06_046 | gi\|399207673\|gb\|AFP33367.1\| | 63.14 | 2.00E-121 | polygalacturonase 6 [Apolygus lucorum] |
| XT5K-14_B06_046 | gi\|145098373\|gb\|ABD63920.1\| | 52.1 | 2.00E-83 | polygalacturonase PG1 [Lygus lineolaris] |
| XT5K-14_B06_046 | gi\|409150895\|gb\|AFV15473.1\| | 49.04 | 2.00E-80 | polygalacturonase PG1 [Apolygus lucorum] |
| XT5K-43_H05_033 | gi\|399207673\|gb\|AFP33367.1\| | 65.91 | 8.00E-117 | polygalacturonase 6 [Apolygus lucorum] |
| XT5K-43_H05_033 | gi\|145098373\|gb\|ABD63920.1\| | 51.66 | 3.00E-80 | polygalacturonase PG1 [Lygus lineolaris] |
| XT5K-43_H05_033 | gi\|409150895\|gb\|AFV15473.1\| | 50.97 | 1.00E-78 | polygalacturonase PG1 [Apolygus lucorum] |
| Contig549 | gi\|145098373\|gb\|ABD63920.1\| | 89.3 | 2.00E-151 | polygalacturonase PG1 [Lygus lineolaris] |
| Contig549 | gi\|399207673\|gb\|AFP33367.1\| | 64.85 | 8.00E-101 | polygalacturonase 6 [Apolygus lucorum] |
| Contig549 | gi\|399207671\|gb\|AFP33366.1\| | 57.59 | 3.00E-94 | polygalacturonase 5 [Apolygus lucorum] |
| A08_XT5K-1 | gi\|399207673\|gb\|AFP33367.1\| | 69.39 | 1.00E-127 | polygalacturonase 6 [Apolygus lucorum] |
| A08_XT5K-1 | gi\|145098373\|gb\|ABD63920.1\| | 62.93 | 8.00E-97 | polygalacturonase PG1 [Lygus lineolaris] |
| A08_XT5K-1 | gi\|399207657\|gb\|AFP33359.1\| | 50.96 | 8.00E-74 | polygalacturonase 1 [Apolygus lucorum] |
| XT5K-27_G02_004 | gi\|399207673\|gb\|AFP33367.1\| | 56.73 | 8.00E-117 | polygalacturonase 6 [Apolygus lucorum] |
| XT5K-27_G02_004 | gi\|145098373\|gb\|ABD63920.1\| | 45.76 | 5.00E-78 | polygalacturonase PG1 [Lygus lineolaris] |
| XT5K-27_G02_004 | gi\|409150895\|gb\|AFV15473.1\| | 42.65 | 2.00E-69 | polygalacturonase PG1 [Apolygus lucorum] |
| XT5K-14_B11_093 | gi\|399207673\|gb\|AFP33367.1\| | 48.24 | 2.00E-20 | polygalacturonase 6 [Apolygus lucorum] |

| | | | | |
|---|---|---|---|---|
| XT5K-14_B11_093 | gi|145098373|gb|ABD63920.1| | 33.33 | 1.00E-11 | polygalacturonase PG1 [Lygus lineolaris] |
| XT5K-14_B11_093 | gi|409150895|gb|AFV15473.1| | 36.27 | 1.00E-04 | polygalacturonase PG1 [Apolygus lucorum] |
| XT5K-14_B12_094 | gi|399207673|gb|AFP33367.1| | 90.91 | 0 | polygalacturonase 6 [Apolygus lucorum] |
| XT5K-14_B12_094 | gi|145098373|gb|ABD63920.1| | 57.88 | 1.00E-116 | polygalacturonase PG1 [Lygus lineolaris] |
| XT5K-14_B12_094 | gi|399207667|gb|AFP33364.1| | 52.72 | 7.00E-95 | polygalacturonase 14 [Apolygus lucorum] |
| XT5K-40_A11_095 | gi|399207673|gb|AFP33367.1| | 61.52 | 8.00E-117 | polygalacturonase 6 [Apolygus lucorum] |
| XT5K-40_A11_095 | gi|145098373|gb|ABD63920.1| | 53.67 | 6.00E-65 | polygalacturonase PG1 [Lygus lineolaris] |
| XT5K-40_A11_095 | gi|409150895|gb|AFV15473.1| | 49.11 | 4.00E-59 | polygalacturonase PG1 [Apolygus lucorum] |
| H05_XT5K-1 | gi|399207659|gb|AFP33360.1| | 45.61 | 0.5 | polygalacturonase 2 [Apolygus lucorum] |
| H05_XT5K-1 | gi|399207669|gb|AFP33365.1| | 74.32 | 9.00E-170 | polygalacturonase 4 [Apolygus lucorum] |
| H05_XT5K-1 | gi|409150911|gb|AFV15475.1| | 74.32 | 2.00E-169 | polygalacturonase PG2-2 [Apolygus lucorum] |
| XT5K-32_G04_020 | gi|399207659|gb|AFP33360.1| | 98.01 | 1.00E-174 | polygalacturonase 2 [Apolygus lucorum] |
| XT5K-32_G04_020 | gi|409150911|gb|AFV15475.1| | 80.56 | 2.00E-144 | polygalacturonase PG2-2 [Apolygus lucorum] |
| XT5K-32_G04_020 | gi|145098420|gb|ABD63921.1| | 75.38 | 6.00E-140 | polygalacturonase PG2 [Lygus lineolaris] |
| XT5K-35_E04_024 | gi|399207659|gb|AFP33360.1| | 81.54 | 0 | polygalacturonase 2 [Apolygus lucorum] |
| XT5K-35_E04_024 | gi|409150911|gb|AFV15475.1| | 69.71 | 5.00E-171 | polygalacturonase PG2-2 [Apolygus lucorum] |
| XT5K-35_E04_024 | gi|399207669|gb|AFP33365.1| | 69.43 | 8.00E-170 | polygalacturonase 4 [Apolygus lucorum] |
| XT5K-52_B06_046 | gi|399207659|gb|AFP33360.1| | 97.27 | 0 | polygalacturonase 2 [Apolygus lucorum] |
| XT5K-52_B06_046 | gi|409150911|gb|AFV15475.1| | 76.53 | 2.00E-164 | polygalacturonase PG2-2 [Apolygus lucorum] |
| XT5K-52_B06_046 | gi|399207669|gb|AFP33365.1| | 76.19 | 2.00E-163 | polygalacturonase 4 [Apolygus lucorum] |
| Contig10 | gi|399207669|gb|AFP33365.1| | 96.17 | 0 | polygalacturonase 4 [Apolygus lucorum] |
| Contig10 | gi|409150911|gb|AFV15475.1| | 96.17 | 0 | polygalacturonase PG2-2 [Apolygus lucorum] |
| Contig10 | gi|145098420|gb|ABD63921.1| | 80.55 | 0 | polygalacturonase PG2 [Lygus lineolaris] |
| Contig353 | gi|399207675|gb|AFP33368.1| | 99.44 | 0 | polygalacturonase 8 [Apolygus lucorum] |
| Contig353 | gi|667527577|gb|KFA55692.1| | 53.22 | 7.00E-89 | hypothetical protein S40293_05245 [Stachybotrys chartarum IBT 40293] |
| Contig353 | gi|667720092|gb|KFA62413.1| | 52.88 | 5.00E-88 | hypothetical protein S40285_06406 [Stachybotrys chlorohalonata IBT 40285] |
| XT5K-25_E07_055 | gi|409150911|gb|AFV15475.1| | 92.2 | 0 | polygalacturonase PG2-2 [Apolygus lucorum] |
| XT5K-25_E07_055 | gi|399207669|gb|AFP33365.1| | 90.75 | 0 | polygalacturonase 4 [Apolygus lucorum] |
| XT5K-25_E07_055 | gi|145098420|gb|ABD63921.1| | 71.62 | 0 | polygalacturonase PG2 [Lygus lineolaris] |
| H12_XT5K-1 | gi|399207669|gb|AFP33365.1| | 86.29 | 2.00E-66 | polygalacturonase 4 [Apolygus lucorum] |
| H12_XT5K-1 | gi|409150911|gb|AFV15475.1| | 83.06 | 7.00E-63 | polygalacturonase PG2-2 [Apolygus lucorum] |

| | | | | |
|---|---|---|---|---|
| H12_XT5K-1 | gi\|145098420\|gb\|ABD63921.1\| | 71.2 | 2.00E-55 | polygalacturonase PG2 [Lygus lineolaris] |
| XT5K-54_C01_011 | gi\|399207669\|gb\|AFP33365.1\| | 96.21 | 0 | polygalacturonase 4 [Apolygus lucorum] |
| XT5K-54_C01_011 | gi\|409150911\|gb\|AFV15475.1\| | 94.83 | 0 | polygalacturonase PG2-2 [Apolygus lucorum] |
| XT5K-54_C01_011 | gi\|145098420\|gb\|ABD63921.1\| | 81.31 | 4.00E-170 | polygalacturonase PG2 [Lygus lineolaris] |
| Contig125 | gi\|399207657\|gb\|AFP33359.1\| | 59.84 | 4.00E-96 | polygalacturonase 1 [Apolygus lucorum] |
| Contig125 | gi\|399207677\|gb\|AFP33369.1\| | 58.9 | 8.00E-92 | polygalacturonase 9 [Apolygus lucorum] |
| Contig125 | gi\|409150895\|gb\|AFV15473.1\| | 57.63 | 1.00E-90 | polygalacturonase PG1 [Apolygus lucorum] |
| Contig249 | gi\|399207657\|gb\|AFP33359.1\| | 60.23 | 7.00E-97 | polygalacturonase 1 [Apolygus lucorum] |
| Contig249 | gi\|399207661\|gb\|AFP33361.1\| | 61.74 | 7.00E-90 | polygalacturonase 10 [Apolygus lucorum] |
| Contig249 | gi\|399207677\|gb\|AFP33369.1\| | 58.96 | 3.00E-89 | polygalacturonase 9 [Apolygus lucorum] |
| XT5K-13_E04_024 | gi\|399207657\|gb\|AFP33359.1\| | 61.01 | 8.00E-123 | polygalacturonase 1 [Apolygus lucorum] |
| XT5K-13_E04_024 | gi\|399207677\|gb\|AFP33369.1\| | 60 | 6.00E-115 | polygalacturonase 9 [Apolygus lucorum] |
| XT5K-13_E04_024 | gi\|409150895\|gb\|AFV15473.1\| | 59.02 | 6.00E-113 | polygalacturonase PG1 [Apolygus lucorum] |
| XT5K-13_E11_087 | gi\|399207657\|gb\|AFP33359.1\| | 58.98 | 1.00E-121 | polygalacturonase 1 [Apolygus lucorum] |
| XT5K-13_E11_087 | gi\|399207677\|gb\|AFP33369.1\| | 58.41 | 3.00E-112 | polygalacturonase 9 [Apolygus lucorum] |
| XT5K-13_E11_087 | gi\|399207661\|gb\|AFP33361.1\| | 61.01 | 2.00E-110 | polygalacturonase 10 [Apolygus lucorum] |
| XT5K-15_A07_063 | gi\|399207657\|gb\|AFP33359.1\| | 60.94 | 5.00E-112 | polygalacturonase 1 [Apolygus lucorum] |
| XT5K-15_A07_063 | gi\|399207677\|gb\|AFP33369.1\| | 55.49 | 2.00E-108 | polygalacturonase 9 [Apolygus lucorum] |
| XT5K-15_A07_063 | gi\|409150895\|gb\|AFV15473.1\| | 54.57 | 2.00E-106 | polygalacturonase PG1 [Apolygus lucorum] |
| XT5K-20_F05_037 | gi\|399207657\|gb\|AFP33359.1\| | 60.38 | 3.00E-98 | polygalacturonase 1 [Apolygus lucorum] |
| XT5K-20_F05_037 | gi\|399207661\|gb\|AFP33361.1\| | 61.07 | 4.00E-90 | polygalacturonase 10 [Apolygus lucorum] |
| XT5K-20_F05_037 | gi\|399207677\|gb\|AFP33369.1\| | 58.96 | 6.00E-90 | polygalacturonase 9 [Apolygus lucorum] |
| XT5K-35_E03_023 | gi\|399207657\|gb\|AFP33359.1\| | 61.64 | 9.00E-118 | polygalacturonase 1 [Apolygus lucorum] |
| XT5K-35_E03_023 | gi\|399207677\|gb\|AFP33369.1\| | 55.49 | 1.00E-110 | polygalacturonase 9 [Apolygus lucorum] |
| XT5K-35_E03_023 | gi\|409150895\|gb\|AFV15473.1\| | 54.3 | 3.00E-108 | polygalacturonase PG1 [Apolygus lucorum] |
| XT5K-35_H12_082 | gi\|399207657\|gb\|AFP33359.1\| | 61.27 | 7.00E-122 | polygalacturonase 1 [Apolygus lucorum] |
| XT5K-35_H12_082 | gi\|399207677\|gb\|AFP33369.1\| | 60.6 | 9.00E-115 | polygalacturonase 9 [Apolygus lucorum] |
| XT5K-35_H12_082 | gi\|409150895\|gb\|AFV15473.1\| | 59.6 | 1.00E-112 | polygalacturonase PG1 [Apolygus lucorum] |
| XT5K-38_C10_076 | gi\|399207657\|gb\|AFP33359.1\| | 57.74 | 8.00E-109 | polygalacturonase 1 [Apolygus lucorum] |
| XT5K-38_C10_076 | gi\|399207677\|gb\|AFP33369.1\| | 56.76 | 2.00E-100 | polygalacturonase 9 [Apolygus lucorum] |
| XT5K-38_C10_076 | gi\|399207661\|gb\|AFP33361.1\| | 59.15 | 2.00E-99 | polygalacturonase 10 [Apolygus lucorum] |

| | | | | |
|---|---|---|---|---|
| XT5K-51_C07_059 | gi\|399207657\|gb\|AFP33359.1\| | 61.59 | 5.00E-117 | polygalacturonase 1 [Apolygus lucorum] |
| XT5K-51_C07_059 | gi\|399207677\|gb\|AFP33369.1\| | 59.86 | 4.00E-107 | polygalacturonase 9 [Apolygus lucorum] |
| XT5K-51_C07_059 | gi\|399207661\|gb\|AFP33361.1\| | 61.59 | 6.00E-106 | polygalacturonase 10 [Apolygus lucorum] |
| XT_G02_004 | gi\|399207677\|gb\|AFP33369.1\| | 60.34 | 5.00E-107 | polygalacturonase 9 [Apolygus lucorum] |
| XT_G02_004 | gi\|409150895\|gb\|AFV15473.1\| | 59.31 | 6.00E-105 | polygalacturonase PG1 [Apolygus lucorum] |
| XT_G02_004 | gi\|399207663\|gb\|AFP33362.1\| | 58.1 | 5.00E-97 | polygalacturonase 11 [Apolygus lucorum] |
| XT5K-9_D01_009 | gi\|399207675\|gb\|AFP33368.1\| | 87.38 | 1.00E-158 | polygalacturonase 8 [Apolygus lucorum] |
| XT5K-9_D01_009 | gi\|667527577\|gb\|KFA55692.1\| | 51.69 | 6.00E-57 | hypothetical protein S40293_05245 [Stachybotrys chartarum IBT 40293] |
| XT5K-9_D01_009 | gi\|667720092\|gb\|KFA62413.1\| | 51.69 | 1.00E-56 | hypothetical protein S40285_06406 [Stachybotrys chlorohalonata IBT 40285] |
| XT5K-32_E11_087 | gi\|399207677\|gb\|AFP33369.1\| | 78.45 | 0 | polygalacturonase 9 [Apolygus lucorum] |
| XT5K-32_E11_087 | gi\|409150895\|gb\|AFV15473.1\| | 78.16 | 0 | polygalacturonase PG1 [Apolygus lucorum] |
| XT5K-32_E11_087 | gi\|145098425\|gb\|ABD63922.1\| | 59.88 | 3.00E-143 | polygalacturonase PG3 [Lygus lineolaris] |
| XT5K-44_C03_027 | gi\|399207677\|gb\|AFP33369.1\| | 81.65 | 2.00E-149 | polygalacturonase 9 [Apolygus lucorum] |
| XT5K-44_C03_027 | gi\|409150895\|gb\|AFV15473.1\| | 80.9 | 1.00E-147 | polygalacturonase PG1 [Apolygus lucorum] |
| XT5K-44_C03_027 | gi\|145098425\|gb\|ABD63922.1\| | 62.02 | 1.00E-111 | polygalacturonase PG3 [Lygus lineolaris] |
| XT5K-44_H04_018 | gi\|145098425\|gb\|ABD63922.1\| | 90.97 | 0 | polygalacturonase PG3 [Lygus lineolaris] |
| XT5K-44_H04_018 | gi\|399207677\|gb\|AFP33369.1\| | 54.8 | 7.00E-111 | polygalacturonase 9 [Apolygus lucorum] |
| XT5K-44_H04_018 | gi\|409150895\|gb\|AFV15473.1\| | 54.49 | 4.00E-110 | polygalacturonase PG1 [Apolygus lucorum] |
| XT5K-26_B02_014 | gi\|399207661\|gb\|AFP33361.1\| | 83.45 | 4.00E-163 | polygalacturonase 10 [Apolygus lucorum] |
| XT5K-26_B02_014 | gi\|399207677\|gb\|AFP33369.1\| | 65.64 | 8.00E-117 | polygalacturonase 9 [Apolygus lucorum] |
| XT5K-26_B02_014 | gi\|409150895\|gb\|AFV15473.1\| | 65.64 | 3.00E-116 | polygalacturonase PG1 [Apolygus lucorum] |
| XT5K-29_E09_071 | gi\|399207661\|gb\|AFP33361.1\| | 93.48 | 2.00E-173 | polygalacturonase 10 [Apolygus lucorum] |
| XT5K-29_E09_071 | gi\|399207657\|gb\|AFP33359.1\| | 91.76 | 4.00E-173 | polygalacturonase 1 [Apolygus lucorum] |
| XT5K-29_E09_071 | gi\|399207677\|gb\|AFP33369.1\| | 72.36 | 2.00E-126 | polygalacturonase 9 [Apolygus lucorum] |
| XT5K-29_F02_006 | gi\|399207657\|gb\|AFP33359.1\| | 90.49 | 3.00E-172 | polygalacturonase 1 [Apolygus lucorum] |
| XT5K-29_F02_006 | gi\|399207661\|gb\|AFP33361.1\| | 78.18 | 5.00E-145 | polygalacturonase 10 [Apolygus lucorum] |
| XT5K-29_F02_006 | gi\|399207677\|gb\|AFP33369.1\| | 63.18 | 1.00E-107 | polygalacturonase 9 [Apolygus lucorum] |
| xt5k-33_B08_062 | gi\|399207657\|gb\|AFP33359.1\| | 98.68 | 0 | polygalacturonase 1 [Apolygus lucorum] |
| xt5k-33_B08_062 | gi\|399207661\|gb\|AFP33361.1\| | 88.78 | 0 | polygalacturonase 10 [Apolygus lucorum] |
| xt5k-33_B08_062 | gi\|399207677\|gb\|AFP33369.1\| | 69.02 | 4.00E-128 | polygalacturonase 9 [Apolygus lucorum] |
| Contig278 | gi\|399207677\|gb\|AFP33369.1\| | 71.6 | 3.00E-164 | polygalacturonase 9 [Apolygus lucorum] |

| | | | | |
|---|---|---|---|---|
| Contig278 | gi\|409150895\|gb\|AFV15473.1\| | 71 | 3.00E-163 | polygalacturonase PG1 [Apolygus lucorum] |
| Contig278 | gi\|399207657\|gb\|AFP33359.1\| | 64.4 | 1.00E-133 | polygalacturonase 1 [Apolygus lucorum] |
| Contig379 | gi\|399207677\|gb\|AFP33369.1\| | 47.18 | 6.00E-73 | polygalacturonase 9 [Apolygus lucorum] |
| Contig379 | gi\|399207673\|gb\|AFP33367.1\| | 52.52 | 2.00E-71 | polygalacturonase 6 [Apolygus lucorum] |
| Contig379 | gi\|409150895\|gb\|AFV15473.1\| | 45.97 | 2.00E-71 | polygalacturonase PG1 [Apolygus lucorum] |
| XT5K-3_C01_011 | gi\|399207677\|gb\|AFP33369.1\| | 69.25 | 2.00E-144 | polygalacturonase 9 [Apolygus lucorum] |
| XT5K-3_C01_011 | gi\|409150895\|gb\|AFV15473.1\| | 68.01 | 5.00E-142 | polygalacturonase PG1 [Apolygus lucorum] |
| XT5K-3_C01_011 | gi\|399207657\|gb\|AFP33359.1\| | 65.98 | 2.00E-124 | polygalacturonase 1 [Apolygus lucorum] |
| XT5K-10_D04_026 | gi\|409150895\|gb\|AFV15473.1\| | 51.16 | 6.00E-100 | polygalacturonase PG1 [Apolygus lucorum] |
| XT5K-10_D04_026 | gi\|145098425\|gb\|ABD63922.1\| | 46.26 | 5.00E-81 | polygalacturonase PG3 [Lygus lineolaris] |
| XT5K-10_D04_026 | gi\|399207657\|gb\|AFP33359.1\| | 49.31 | 1.00E-78 | polygalacturonase 1 [Apolygus lucorum] |
| XT5K-11_C04_028 | gi\|399207677\|gb\|AFP33369.1\| | 71.54 | 9.00E-121 | polygalacturonase 9 [Apolygus lucorum] |
| XT5K-11_C04_028 | gi\|409150895\|gb\|AFV15473.1\| | 70.04 | 2.00E-118 | polygalacturonase PG1 [Apolygus lucorum] |
| XT5K-11_C04_028 | gi\|399207657\|gb\|AFP33359.1\| | 64.34 | 8.00E-103 | polygalacturonase 1 [Apolygus lucorum] |
| XT5K-50_F10_070 | gi\|399207677\|gb\|AFP33369.1\| | 67.19 | 3.00E-134 | polygalacturonase 9 [Apolygus lucorum] |
| XT5K-50_F10_070 | gi\|409150895\|gb\|AFV15473.1\| | 65.94 | 6.00E-132 | polygalacturonase PG1 [Apolygus lucorum] |
| XT5K-50_F10_070 | gi\|399207657\|gb\|AFP33359.1\| | 64.21 | 1.00E-114 | polygalacturonase 1 [Apolygus lucorum] |
| XT5K-43_G06_036 | gi\|399207677\|gb\|AFP33369.1\| | 82.15 | 9.00E-170 | polygalacturonase 9 [Apolygus lucorum] |
| XT5K-43_G06_036 | gi\|409150895\|gb\|AFV15473.1\| | 81.48 | 5.00E-168 | polygalacturonase PG1 [Apolygus lucorum] |
| XT5K-43_G06_036 | gi\|399207657\|gb\|AFP33359.1\| | 65.36 | 2.00E-133 | polygalacturonase 1 [Apolygus lucorum] |
| XT5K-9_C06_044 | gi\|145098425\|gb\|ABD63922.1\| | 89.64 | 5.00E-123 | polygalacturonase PG3 [Lygus lineolaris] |
| XT5K-9_C06_044 | gi\|399207657\|gb\|AFP33359.1\| | 62.98 | 6.00E-73 | polygalacturonase 1 [Apolygus lucorum] |
| XT5K-9_C06_044 | gi\|399207677\|gb\|AFP33369.1\| | 59.9 | 3.00E-71 | polygalacturonase 9 [Apolygus lucorum] |
| XT5K-9_D11_089 | gi\|145098425\|gb\|ABD63922.1\| | 80.19 | 1.00E-45 | polygalacturonase PG3 [Lygus lineolaris] |
| XT5K-9_D11_089 | gi\|399207663\|gb\|AFP33362.1\| | 51 | 1.00E-18 | polygalacturonase 11 [Apolygus lucorum] |
| XT5K-9_D11_089 | gi\|183181578\|gb\|ACC44844.1\| | 47.17 | 1.00E-17 | polygalacturonase [Lygus hesperus] |
| XT5K-9_F12_086 | gi\|399207657\|gb\|AFP33359.1\| | 47.37 | 1.00E-55 | polygalacturonase 1 [Apolygus lucorum] |
| XT5K-9_F12_086 | gi\|399207661\|gb\|AFP33361.1\| | 45.4 | 3.00E-41 | polygalacturonase 10 [Apolygus lucorum] |
| XT5K-9_F12_086 | gi\|399207677\|gb\|AFP33369.1\| | 36.03 | 1.00E-22 | polygalacturonase 9 [Apolygus lucorum] |
| XT5K-9_D01_009 | gi\|399207675\|gb\|AFP33368.1\| | 87.38 | 1.00E-158 | polygalacturonase 8 [Apolygus lucorum] |
| XT5K-9_D01_009 | gi\|667527577\|gb\|KFA55692.1\| | 51.69 | 6.00E-57 | hypothetical protein S40293_05245 [Stachybotrys chartarum IBT 40293] |

| | | | | |
|---|---|---|---|---|
| XT5K-9_D01_009 | gi\|667720092\|gb\|KFA62413.1\| | 51.69 | 1.00E-56 | hypothetical protein S40285_06406 [Stachybotrys chlorohalonata IBT 40285] |
| XT5K-12_D01_009 | gi\|399207675\|gb\|AFP33368.1\| | 96.54 | 5.00E-175 | polygalacturonase 8 [Apolygus lucorum] |
| XT5K-12_D01_009 | gi\|667527577\|gb\|KFA55692.1\| | 54.47 | 1.00E-64 | hypothetical protein S40293_05245 [Stachybotrys chartarum IBT 40293] |
| XT5K-12_D01_009 | gi\|667720092\|gb\|KFA62413.1\| | 54.04 | 3.00E-64 | hypothetical protein S40285_06406 [Stachybotrys chlorohalonata IBT 40285] |
| XT5K-13_C07_059 | gi\|399207675\|gb\|AFP33368.1\| | 98.88 | 1.00E-163 | polygalacturonase 8 [Apolygus lucorum] |
| XT5K-13_C07_059 | gi\|667527577\|gb\|KFA55692.1\| | 56.73 | 3.00E-59 | hypothetical protein S40293_05245 [Stachybotrys chartarum IBT 40293] |
| XT5K-13_C07_059 | gi\|667720092\|gb\|KFA62413.1\| | 56.25 | 8.00E-59 | hypothetical protein S40285_06406 [Stachybotrys chlorohalonata IBT 40285] |
| XT5K-35_F08_054 | gi\|399207675\|gb\|AFP33368.1\| | 98.21 | 5.00E-173 | polygalacturonase 8 [Apolygus lucorum] |
| XT5K-35_F08_054 | gi\|183181578\|gb\|ACC44844.1\| | 51.07 | 4.00E-63 | polygalacturonase [Lygus hesperus] |
| XT5K-35_F08_054 | gi\|667527577\|gb\|KFA55692.1\| | 55.5 | 7.00E-63 | hypothetical protein S40293_05245 [Stachybotrys chartarum IBT 40293] |
| XT5K-43_B05_045 | gi\|399207675\|gb\|AFP33368.1\| | 98.88 | 9.00E-165 | polygalacturonase 8 [Apolygus lucorum] |
| XT5K-43_B05_045 | gi\|667527577\|gb\|KFA55692.1\| | 56.46 | 1.00E-59 | hypothetical protein S40293_05245 [Stachybotrys chartarum IBT 40293] |
| XT5K-43_B05_045 | gi\|667720092\|gb\|KFA62413.1\| | 55.98 | 3.00E-59 | hypothetical protein S40285_06406 [Stachybotrys chlorohalonata IBT 40285] |
| Contig486 | gi\|399207675\|gb\|AFP33368.1\| | 66.47 | 3.00E-138 | polygalacturonase 8 [Apolygus lucorum] |
| Contig486 | gi\|183181578\|gb\|ACC44844.1\| | 48.65 | 3.00E-74 | polygalacturonase [Lygus hesperus] |
| Contig486 | gi\|399207667\|gb\|AFP33364.1\| | 48.41 | 1.00E-71 | polygalacturonase 14 [Apolygus lucorum] |
| Contig504 | gi\|399207665\|gb\|AFP33363.1\| | 81.74 | 1.00E-180 | polygalacturonase 12 [Apolygus lucorum] |
| Contig504 | gi\|399207667\|gb\|AFP33364.1\| | 81.29 | 4.00E-180 | polygalacturonase 14 [Apolygus lucorum] |
| Contig504 | gi\|183181578\|gb\|ACC44844.1\| | 79.59 | 3.00E-174 | polygalacturonase [Lygus hesperus] |
| XT5K-5_B01_013 | gi\|399207675\|gb\|AFP33368.1\| | 64.95 | 1.00E-120 | polygalacturonase 8 [Apolygus lucorum] |
| XT5K-5_B01_013 | gi\|183181578\|gb\|ACC44844.1\| | 47.46 | 7.00E-63 | polygalacturonase [Lygus hesperus] |
| XT5K-5_B01_013 | gi\|129938\|gb\|P26215.1\| | 50.4 | 3.00E-62 | RecName: Full=Polygalacturonase; Short=PG; AltName: Full=Pectinase; Flags: Precursor [Bipolaris zeicola] |
| XT5K-7_B09_077 | gi\|399207675\|gb\|AFP33368.1\| | 66.67 | 1.00E-125 | polygalacturonase 8 [Apolygus lucorum] |
| XT5K-7_B09_077 | gi\|183181578\|gb\|ACC44844.1\| | 47.18 | 4.00E-65 | polygalacturonase [Lygus hesperus] |
| XT5K-7_B09_077 | gi\|475676944\|gb\|EMT73962.1\| | 48.52 | 5.00E-64 | hypothetical protein FOC4_g10000234 [Fusarium oxysporum f. sp. cubense race 4] |
| XT5K-10_B06_046 | gi\|399207675\|gb\|AFP33368.1\| | 65.43 | 6.00E-61 | polygalacturonase 8 [Apolygus lucorum] |
| XT5K-10_B06_046 | gi\|323332833\|gb\|EGA74237.1\| | 50.27 | 7.00E-37 | Pgu1p [Saccharomyces cerevisiae AWRI796] |
| XT5K-10_B06_046 | gi\|398365647\|gb\|NP_012687.1\| | 50.27 | 8.00E-37 | Pgu1p [Saccharomyces cerevisiae S288c] |
| XT5K-34_D03_025 | gi\|399207675\|gb\|AFP33368.1\| | 66.67 | 3.00E-120 | polygalacturonase 8 [Apolygus lucorum] |
| XT5K-34_D03_025 | gi\|183181578\|gb\|ACC44844.1\| | 47.46 | 7.00E-63 | polygalacturonase [Lygus hesperus] |
| XT5K-34_D03_025 | gi\|129938\|gb\|P26215.1\| | 50.81 | 2.00E-62 | RecName: Full=Polygalacturonase; Short=PG; AltName: Full=Pectinase; Flags: Precursor [Bipolaris zeicola] |

| | | | | |
|---|---|---|---|---|
| XT5K-35_B03_029 | gi\|399207675\|gb\|AFP33368.1\| | 66.79 | 5.00E-119 | polygalacturonase 8 [Apolygus lucorum] |
| XT5K-35_B03_029 | gi\|183181578\|gb\|ACC44844.1\| | 47.46 | 1.00E-62 | polygalacturonase [Lygus hesperus] |
| XT5K-35_B03_029 | gi\|129938\|gb\|P26215.1\| | 50.4 | 8.00E-62 | RecName: Full=Polygalacturonase; Short=PG; AltName: Full=Pectinase; Flags: Precursor [Bipolaris zeicola] |
| XT5K-37_G03_019 | gi\|399207675\|gb\|AFP33368.1\| | 67.66 | 2.00E-102 | polygalacturonase 8 [Apolygus lucorum] |
| XT5K-37_G03_019 | gi\|667527577\|gb\|KFA55692.1\| | 47.41 | 8.00E-54 | hypothetical protein S40293_05245 [Stachybotrys chartarum IBT 40293] |
| XT5K-37_G03_019 | gi\|183181578\|gb\|ACC44844.1\| | 51.02 | 3.00E-52 | polygalacturonase [Lygus hesperus] |
| XT5K-50_E08_056 | gi\|399207675\|gb\|AFP33368.1\| | 65.88 | 1.00E-132 | polygalacturonase 8 [Apolygus lucorum] |
| XT5K-50_E08_056 | gi\|183181578\|gb\|ACC44844.1\| | 46.44 | 1.00E-67 | polygalacturonase [Lygus hesperus] |
| XT5K-50_E08_056 | gi\|129938\|gb\|P26215.1\| | 50.53 | 8.00E-66 | RecName: Full=Polygalacturonase; Short=PG; AltName: Full=Pectinase; Flags: Precursor [Bipolaris zeicola] |
| XT5K-54_B09_077 | gi\|399207675\|gb\|AFP33368.1\| | 66.32 | 7.00E-124 | polygalacturonase 8 [Apolygus lucorum] |
| XT5K-54_B09_077 | gi\|183181578\|gb\|ACC44844.1\| | 47.22 | 6.00E-65 | polygalacturonase [Lygus hesperus] |
| XT5K-54_B09_077 | gi\|129938\|gb\|P26215.1\| | 50.19 | 2.00E-63 | RecName: Full=Polygalacturonase; Short=PG; AltName: Full=Pectinase; Flags: Precursor [Bipolaris zeicola] |
| XT5K-4_F06_038 | gi\|399207663\|gb\|AFP33362.1\| | 60.19 | 3.00E-32 | polygalacturonase 11 [Apolygus lucorum] |
| XT5K-4_F06_038 | gi\|183181580\|gb\|ACC44845.1\| | 61.62 | 4.00E-31 | polygalacturonase [Lygus hesperus] |
| XT5K-4_F06_038 | gi\|183181278\|gb\|ACC44798.1\| | 61.62 | 5.00E-31 | polygalacturonase [Lygus hesperus] |
| Contig458 | gi\|183181580\|gb\|ACC44845.1\| | 52.71 | 9.00E-95 | polygalacturonase [Lygus hesperus] |
| Contig458 | gi\|183181578\|gb\|ACC44844.1\| | 52.41 | 2.00E-94 | polygalacturonase [Lygus hesperus] |
| Contig458 | gi\|183181278\|gb\|ACC44798.1\| | 50.3 | 1.00E-93 | polygalacturonase [Lygus hesperus] |
| Contig552 | gi\|183181578\|gb\|ACC44844.1\| | 49.22 | 1.00E-87 | polygalacturonase [Lygus hesperus] |
| Contig552 | gi\|183181580\|gb\|ACC44845.1\| | 48.91 | 2.00E-87 | polygalacturonase [Lygus hesperus] |
| Contig552 | gi\|183181278\|gb\|ACC44798.1\| | 49.22 | 5.00E-87 | polygalacturonase [Lygus hesperus] |
| XT5K-3_H04_018 | gi\|183181278\|gb\|ACC44798.1\| | 48.82 | 2.00E-71 | polygalacturonase [Lygus hesperus] |
| XT5K-3_H04_018 | gi\|183181580\|gb\|ACC44845.1\| | 48.82 | 2.00E-71 | polygalacturonase [Lygus hesperus] |
| XT5K-3_H04_018 | gi\|183181578\|gb\|ACC44844.1\| | 48.48 | 3.00E-71 | polygalacturonase [Lygus hesperus] |
| XT5K-31_B12_094 | gi\|183181278\|gb\|ACC44798.1\| | 45.37 | 2.00E-75 | polygalacturonase [Lygus hesperus] |
| XT5K-31_B12_094 | gi\|183181578\|gb\|ACC44844.1\| | 45.05 | 6.00E-75 | polygalacturonase [Lygus hesperus] |
| XT5K-31_B12_094 | gi\|183181580\|gb\|ACC44845.1\| | 44.73 | 3.00E-74 | polygalacturonase [Lygus hesperus] |
| XT5K-53_G07_051 | gi\|183181578\|gb\|ACC44844.1\| | 42.96 | 1.00E-63 | polygalacturonase [Lygus hesperus] |
| XT5K-53_G07_051 | gi\|183181580\|gb\|ACC44845.1\| | 42.61 | 2.00E-63 | polygalacturonase [Lygus hesperus] |
| XT5K-53_G07_051 | gi\|183181278\|gb\|ACC44798.1\| | 42.96 | 4.00E-63 | polygalacturonase [Lygus hesperus] |
| XT5K-38_C01_011 | gi\|183181578\|gb\|ACC44844.1\| | 57.44 | 2.00E-71 | polygalacturonase [Lygus hesperus] |

| | | | | |
|---|---|---|---|---|
| XT5K-38_C01_011 | gi\|183181278\|gb\|ACC44798.1\| | 57.02 | 2.00E-71 | polygalacturonase [Lygus hesperus] |
| XT5K-38_C01_011 | gi\|183181580\|gb\|ACC44845.1\| | 56.61 | 4.00E-70 | polygalacturonase [Lygus hesperus] |
| XT5K-40_E09_071 | gi\|183181578\|gb\|ACC44844.1\| | 57.97 | 7.00E-77 | polygalacturonase [Lygus hesperus] |
| XT5K-40_E09_071 | gi\|183181278\|gb\|ACC44798.1\| | 60.22 | 9.00E-77 | polygalacturonase [Lygus hesperus] |
| XT5K-40_E09_071 | gi\|183181580\|gb\|ACC44845.1\| | 57.63 | 2.00E-76 | polygalacturonase [Lygus hesperus] |
| XT5K-14_A05_047 | gi\|399207667\|gb\|AFP33364.1\| | 99.64 | 1.00E-158 | polygalacturonase 14 [Apolygus lucorum] |
| XT5K-14_A05_047 | gi\|399207665\|gb\|AFP33363.1\| | 92.7 | 3.00E-148 | polygalacturonase 12 [Apolygus lucorum] |
| XT5K-14_A05_047 | gi\|183181578\|gb\|ACC44844.1\| | 82.67 | 1.00E-129 | polygalacturonase [Lygus hesperus] |
| XT5K-30_F07_053 | gi\|399207667\|gb\|AFP33364.1\| | 99.14 | 5.00E-148 | polygalacturonase 14 [Apolygus lucorum] |
| XT5K-30_F07_053 | gi\|399207665\|gb\|AFP33363.1\| | 90.99 | 2.00E-135 | polygalacturonase 12 [Apolygus lucorum] |
| XT5K-30_F07_053 | gi\|183181578\|gb\|ACC44844.1\| | 81.55 | 1.00E-115 | polygalacturonase [Lygus hesperus] |
| XT5K-39_B02_014 | gi\|399207663\|gb\|AFP33362.1\| | 88.05 | 2.00E-171 | polygalacturonase 11 [Apolygus lucorum] |
| XT5K-39_B02_014 | gi\|399207657\|gb\|AFP33359.1\| | 58.55 | 3.00E-105 | polygalacturonase 1 [Apolygus lucorum] |
| XT5K-39_B02_014 | gi\|399207677\|gb\|AFP33369.1\| | 59.86 | 8.00E-103 | polygalacturonase 9 [Apolygus lucorum] |
| XT5K-50_H08_050 | gi\|409150895\|gb\|AFV15473.1\| | 57.55 | 2.00E-70 | polygalacturonase PG1 [Apolygus lucorum] |
| XT5K-50_H08_050 | gi\|399207657\|gb\|AFP33359.1\| | 54.88 | 7.00E-68 | polygalacturonase 1 [Apolygus lucorum] |
| XT5K-50_H08_050 | gi\|399207661\|gb\|AFP33361.1\| | 57.28 | 1.00E-63 | polygalacturonase 10 [Apolygus lucorum] |
