# Supplementary material for: Molecular Characterization and Expression Profiles of Polygalacturonase Genes in Apolygus lucorum (Hemiptera: Miridae)
Source: PLoS One. 2015 May 8;10(5):e0126391. doi: 10.1371/journal.pone.0126391 (PMC4425681; doi:10.1371/journal.pone.0126391)
Supplement: S3 Table — (PDF) [file pone.0126391.s006.pdf]

S3 Table: Identities of the PG amino acid sequences from *Apolygus lucorum*.

Names of PG genes	PG1-1	PG1-2	PG1-3	PG2-1	PG2-2	PG3-1	PG3-2	PG3-3	PG3-4	PG3-5	PG4	PG5-1	PG5-2	PG6	AFP33359	AFP33360	AFP33361	AFP33362	AFP33363	AFP33364	AFP33365	AFP33366	AFP33367	AFP33368	AFP33369	AFV15473	AFV15474	AFV15475
PG1-1	ID																											
PG1-2	0.617	ID																										
PG1-3	0.685	0.733	ID																									
PG2-1	0.396	0.409	0.439	ID																								
PG2-2	0.397	0.435	0.44	0.778	ID																							
PG3-1	0.455	0.449	0.441	0.346	0.337	ID																						
PG3-2	0.488	0.461	0.463	0.36	0.357	0.538	ID																					
PG3-3	0.504	0.484	0.501	0.374	0.36	0.565	0.621	ID																				
PG3-4	0.486	0.483	0.48	0.379	0.368	0.557	0.716	0.607	ID																			
PG3-5	0.5	0.461	0.477	0.347	0.362	0.557	0.817	0.641	0.723	ID																		
PG4	0.465	0.495	0.493	0.412	0.412	0.416	0.47	0.426	0.451	0.465	ID																	
PG5-1	0.493	0.463	0.522	0.4	0.38	0.446	0.528	0.494	0.504	0.515	0.476	ID																
PG5-2	0.461	0.472	0.477	0.378	0.38	0.455	0.522	0.461	0.519	0.488	0.45	0.54	ID															
PG6	0.401	0.387	0.376	0.311	0.316	0.36	0.4	0.365	0.39	0.383	0.37	0.445	0.386	ID														
AFP33359	0.504	0.481	0.501	0.377	0.37	0.576	0.627	0.932	0.616	0.652	0.437	0.494	0.463	0.365	ID													
AFP33360	0.39	0.409	0.439	0.989	0.778	0.343	0.355	0.366	0.374	0.342	0.415	0.395	0.373	0.308	0.371	ID												
AFP33361	0.49	0.481	0.498	0.385	0.365	0.571	0.619	0.89	0.602	0.63	0.44	0.491	0.477	0.368	0.834	0.379	ID											
AFP33362	0.473	0.476	0.509	0.403	0.386	0.525	0.659	0.583	0.629	0.718	0.464	0.48	0.457	0.375	0.588	0.395	0.577	ID										
AFP33363	0.493	0.461	0.53	0.389	0.383	0.441	0.545	0.491	0.501	0.535	0.479	0.924	0.529	0.447	0.491	0.387	0.491	0.5	ID									
AFP33364	0.493	0.463	0.519	0.397	0.383	0.444	0.528	0.491	0.504	0.515	0.476	0.985	0.537	0.439	0.491	0.392	0.488	0.477	0.916	ID								
AFP33365	0.397	0.438	0.44	0.773	0.964	0.34	0.357	0.365	0.368	0.362	0.415	0.383	0.377	0.319	0.373	0.773	0.37	0.386	0.386	0.386	ID							
AFP33366	0.372	0.394	0.41	0.46	0.474	0.343	0.331	0.337	0.336	0.331	0.386	0.361	0.337	0.312	0.346	0.463	0.351	0.356	0.364	0.361	0.471	ID						
AFP33367	0.688	0.73	0.98	0.439	0.443	0.447	0.469	0.506	0.488	0.483	0.495	0.525	0.486	0.379	0.506	0.436	0.504	0.52	0.533	0.522	0.443	0.419	ID					
AFP33368	0.423	0.393	0.395	0.306	0.306	0.386	0.389	0.382	0.38	0.379	0.373	0.428	0.394	0.647	0.39	0.306	0.382	0.36	0.426	0.426	0.303	0.304	0.403	ID				
AFP33369	0.502	0.461	0.48	0.35	0.365	0.56	0.822	0.644	0.72	0.982	0.47	0.512	0.488	0.383	0.655	0.344	0.63	0.712	0.532	0.512	0.365	0.331	0.486	0.376	ID			
AFV15473	0.505	0.466	0.483	0.352	0.362	0.552	0.817	0.647	0.715	0.957	0.476	0.509	0.488	0.389	0.652	0.347	0.633	0.718	0.526	0.509	0.362	0.336	0.488	0.387	0.971	ID		
AFV15474	0.413	0.432	0.443	0.756	0.784	0.358	0.373	0.384	0.389	0.362	0.428	0.388	0.356	0.329	0.392	0.759	0.392	0.402	0.391	0.386	0.797	0.494	0.443	0.306	0.365	0.37	ID	
AFV15475	0.395	0.435	0.438	0.773	0.972	0.337	0.354	0.362	0.37	0.36	0.412	0.386	0.382	0.319	0.37	0.773	0.368	0.386	0.388	0.388	0.978	0.474	0.44	0.303	0.362	0.36	0.784	ID
