# Supplementary material for: Molecular Characterization and Expression Profiles of Polygalacturonase Genes in Apolygus lucorum (Hemiptera: Miridae)
Source: PLoS One. 2015 May 8;10(5):e0126391. doi: 10.1371/journal.pone.0126391 (PMC4425681; doi:10.1371/journal.pone.0126391)
Supplement: S4 Table — (PDF) [file pone.0126391.s007.pdf]

S4 Table: Paired Student's t-test of relative PG mRNA expression levels between male and female adults of *Apolygus lucorum*. The data represent the mean±SE of at least 3 repeats normalized relative to β-actin and GAPDH. * P<0.05, ** P<0.01.

PG	1 day old		5 days old		20 days old		30 days old	
	Male	Female	Male	Female	Male	Female	Male	Female
**PG1-1**	0.0009+0.0002	0.0013+0.0005	0.0047+0.0001*	0.0021+0.0001**	0.0073+0.0008	0.0087+0.0044	0.0073+0.0001	0.0076+0.0020
**PG1-2**	0.0115+0.0032	0.0195+0.0058	0.2708+0.0244	0.0602+0.0112**	0.1993+0.0567	0.0728+0.0313	0.1237+0.0413	0.0405+0.0090
**PG1-3**	0.0237+0.0044	0.0265+0.0096	0.2334+0.0429	0.0408+0.0137*	0.3673+0.0868	0.1349+0.0909	0.2395+0.0833	0.0867+0.0058
**PG2-1**	0.0013+0.0006	0.0035+0.0017	0.0201+0.0077	0.0044+0.0020	0.1206+0.0878	0.0236+0.0054	0.0675+0.0257	0.0661+0.0241
**PG2-2**	0.0198+0.0063	0.0255+0.0064	0.2858+0.0317	0.1496+0.0517	0.7046+0.4065	0.1394+0.0787	0.5115+0.1497	0.1358+0.0189
**PG3-1**	0.0116+0.0039	0.0070+0.0029	0.1453+0.0209	0.0279+0.0064**	0.1139+0.0581	0.0266+0.0109	0.0624+0.0207	0.0135+0.0057
**PG3-2**	0.0043+0.0014	0.0042+0.0025	0.0145+0.0029	0.0027+0.0007*	0.0571+0.0225	0.0593+0.0209	0.0562+0.0263	0.0737+0.0407
**PG3-3**	0.0464+0.0207	0.0304+0.0095	0.8375+0.1428	0.2032+0.0500*	0.7994+0.3895	0.6055+0.2357	0.8442+0.2411	0.4450+0.1214
**PG3-4**	0.0121+0.0078	0.0155+0.0023	0.0921+0.0319	0.0404+0.0129	0.1722+0.0199	0.1444+0.0736	0.0740+0.0107	0.0929+0.0042
**PG3-5**	0.0136+0.0043	0.0115+0.0030	0.5042+0.0993	0.1959+0.0661	0.6266+0.2198	0.2735+0.1558	0.5535+0.2103	0.2318+0.0511
**PG4**	0.0238+0.0054	0.0403+0.0141	0.1387+0.0151	0.0366+0.0126**	0.2968+0.0423	0.1327+0.0702	0.2287+0.0997	0.1542+0.0401
**PG5-1**	0.0459+0.0114	0.0283+0.0073	0.5946+0.0243	0.1017+0.0297**	0.5096+0.2883	0.1403+0.0964	0.3892+0.0953	0.1027+0.0312*
**PG5-2**	0.0050+0.0019	0.0071+0.0022	0.0703+0.0215	0.0130+0.0023	0.1298+0.0246	0.0530+0.0156	0.0695+0.0296	0.0451+0.0006
**PG6**	0.0005+0.0004	0.0010+0.0004	0.0138+0.0015	0.0069+0.0016*	0.0192+0.0015	0.0171+0.0072	0.0138+0.0009	0.0097+0.0013
